# Gender and age variations in neuropsychiatric adverse events of cetirizine and levocetirizine: a disproportionality analysis of ICSRs from FAERS and EudraVigilance data

**DOI:** 10.3389/fphar.2025.1737359

**Published:** 2026-01-09

**Authors:** Tingting Yang, Xiaoxiao Wu, Xueliang Yi, Chunlin Xiang, Yinping Yang, Chen Yang, Xiaoxia Zheng, Yuhong Han, Yiping Wang

**Affiliations:** 1 Department of Intensive Care Unit, Sichuan Provincial People’s Hospital East Sichuan Hospital & Dazhou First People’s Hospital, Dazhou, China; 2 Department of Intensive Care Unit, Sichuan Provincial People’s Hospital, University of Electronic Science and Technology of China, Chengdu, China; 3 Department of Emergency, Clinical Medical College and Affliated Hospital of Chengdu University, Chengdu University, Chengdu, China; 4 Department of Critical Care Medicine, The Affiliated Hospital of Southwest Medical University, Luzhou, China

**Keywords:** cetirizine, FAERS, gender and age difference, levocetirizine, neuropsychiatric adverse events

## Abstract

**Background:**

This study analyzed neuropsychiatric adverse drug events (ADEs) associated with cetirizine and levocetirizine using data from the FDA Adverse Event Reporting System (FAERS) and EudraVigilance databases to inform safer clinical use for different age and gender groups.

**Methods:**

We performed disproportionality analyses (ROR, PRR, BCPNN, MGPS) using data from the FAERS (Q1 2004 to Q3 2025) and the EudraVigilance database (January 2002 to December 2025). In addition, stratified analysis was conducted for the top 20 reported ADEs across different sex and age groups.

**Results:**

Somnolence, dizziness, headache, and insomnia are common adverse drug reactions (ADRs) with high disproportionality signals for both cetirizine and levocetirizine. Cetirizine was generally associated with higher signal intensities for depression, anxiety, and drug abuse/dependence compared to levocetirizine, with unique reports of cognitive impairment and migraine. In contrast, levocetirizine showed stronger signals not only for sleep-related ADRs but also for serious psychiatric events, including suicide attempts and suicidal ideation. Among levocetirizine users, febrile convulsions were more frequently reported in males. Additionally, pediatric patients exposed to levocetirizine were reported to have febrile convulsion and epilepsy. In the elderly, reports associated with cetirizine included subarachnoid hemorrhage, transient ischemic attack, and carotid artery occlusion, while those for levocetirizine included hepatic encephalopathy.

**Conclusion:**

These findings highlight the distinct neuropsychiatric risk profiles associated with cetirizine and levocetirizine. This underscores the importance of age- and sex-informed drug selection to optimize their safe use.

## 1 Background

Allergic diseases are among the most prevalent chronic conditions globally. Second-generation antihistamines, the cornerstone of treatment, have raised safety concerns, particularly regarding neuropsychiatric adverse drug events (ADEs) (Nerush et al., 2024). Cetirizine, a selective H1 receptor antagonist, exhibits low lipid solubility and P-glycoprotein efflux, limiting central nervous system penetration and reducing neuropsychiatric risk compared to first-generation agents. Nonetheless, recent studies have reported drowsiness, anxiety, depression, and even hallucinations associated with cetirizine use (Li et al., 2022; Bian et al., 2021; Raju et al., 2022; Hu et al., 2024). Levocetirizine, the active enantiomer of cetirizine, was developed to improve tolerability by optimizing molecular configuration. However, neuropsychiatric ADEs have also been observed with levocetirizine (Li et al., 2022; Bian et al., 2021; Hu et al., 2024).

Clinical pharmacy emphasizes inter-individual pharmacokinetic variability, making precision dosing a key therapeutic goal. Notably, gender and age are the most readily accessible clinical variables. Research has shown that gender markedly affects absorption, distribution, metabolism, and excretion of drugs, making differences in ADEs between genders an important subject warranting in-depth investigation and scientific exploration (Miller, 2014). In pediatrics, distinct physiological characteristics—such as body fluid composition, plasma protein levels, gastric pH, gastric emptying, and immature renal function—affect ADE incidence and presentation (Lu and Rosenbaum, 2014). In the elderly, age-related pharmacokinetic and pharmacodynamic changes, along with comorbidities and polypharmacy, further alter ADE profiles (Drenth-van Maanen et al., 2020; Bellanca et al., 2023). Therefore, the ADE profiles of both cetirizine and levocetirizine—compounds with low metabolic liability and primary renal elimination—are likely modulated by age and sex (Chen, 2008).

Disproportionality analysis, a key pharmacovigilance tool, identifies drug-event associations that occur more frequently than expected by comparing observed and expected reporting rates across the database (Yoshimura et al., 2013). FDA Adverse Event Reporting System (FAERS) and EudraVigilance provide valuable real-world evidence for drug safety surveillance. Through quantitative methods, it facilitates early signal detection and risk assessment, thereby enhancing pharmacovigilance effectiveness (Sakaeda et al., 2013).

This study utilized the data of the FAERS and EudraVigilance databases to evaluate neuropsychiatric ADEs associated with cetirizine and levocetirizine, providing the first comprehensive comparison of their age- and gender-specific differences.

## 2 Methods

### 2.1 Data sources and pre-processing

This disproportionality analysis was conducted and reported following the READUS-PV (REporting of studies Aimed at evaluating Disproportionality in pharmacovigilance Using real-world healthcare Data) statement (Supplementary Appendix 1). Individual case safety reports (ICSRs) were obtained from two pharmacovigilance databases: FAERS for primary analysis and EudraVigilance for external validation. For the FAERS database, we obtained the quarterly data files for cetirizine and levocetirizine from the official U.S. FDA website, covering the period from the first quarter of 2004 to the third quarter of 2025. Data from the EudraVigilance database were extracted on 14 November 2025, and encompass reports from January 2002 to November 2025. Further details regarding both databases are provided in the Supplementary Material.

Data processing followed FDA guidelines. From the DEMO table, we utilized the PRIMARYID, CASEID, and FDA_DT fields to deduplicate reports. For entries sharing the same CASEID, the record with the most recent FDA_DT was retained. In instances where both CASEID and FDA_DT were identical, the record with the largest PRIMARYID was selected. Furthermore, starting from the first quarter of 2019, each quarterly data package included a list of deleted reports. Following the deduplication process, any report whose CASEID appeared in the deletion list was subsequently removed.

To standardize drug nomenclature, we applied the WHO Drug Dictionary (March 2025 version) to all “DRUGNAME” entries in the FAERS dataset. This process involved systematically mapping and normalizing entries potentially related to the target drugs, with manual verification employed to correct any unrecognized names. Subsequent screening and analysis were then conducted based on the standardized generic names for cetirizine and levocetirizine.

**FIGURE 1:**
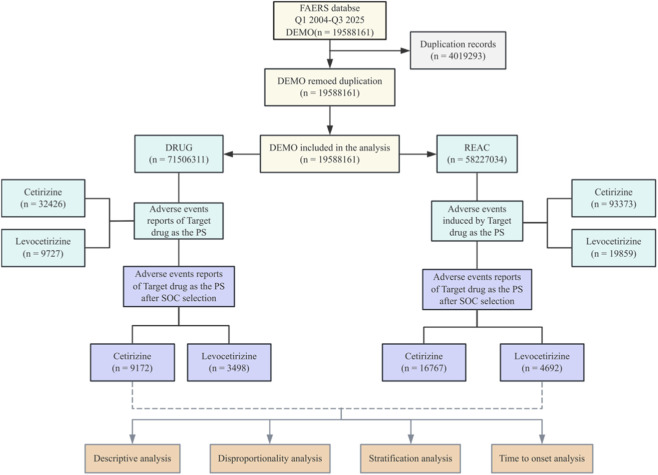
The flow chart of the study of FAERS.

To enhance the accuracy of results and minimize confounding effects, only reports listing the drug as the primary suspect (PS) were included from both the FAERS and EudraVigilance databases. Adverse events (AEs) were categorized using the Medical Dictionary for Regulatory Activities (MedDRA) version 28.1, specifically through System Organ Class (SOC) classification with standardized mapping of preferred terms (PTs) applied consistently across both databases. Based on this SOC framework, the analysis focused on the Nervous System Disorders and Psychiatric Disorders categories. The data processing flow for the FAERS database is detailed in Figure 1, and the corresponding flow for the EudraVigilance database is presented in Supplementary Figure S1.

### 2.2 Disproportionality analysis

Signal detection was performed using disproportionality methods, including Reporting Odds Ratio (ROR), Proportional Reporting Ratio (PRR), Multi-Item Gamma Poisson Shrinker (MGPS), and Bayesian Confidence Propagation Neural Network (BCPNN). Frequency-based methods (ROR/PRR) offer high sensitivity and computational simplicity but are prone to false positives in small samples (Jiang et al., 2024). Bayesian approaches (BCPNN/MGPS) better identify rare events by reducing false positives through statistical correction, albeit with higher computational demands and potential signal delay (Nomura et al., 2015). Based on 2 × 2 contingency tables (Supplementary Table S1), the combined application of these methods may improve detection accuracy by leveraging their complementary strengths. PTs, MedDRA’s core vocabulary, provide standardized and explicit definitions of medical events (Brown, 2004).

### 2.3 Sensitivity analyses

To evaluate the robustness of our primary findings, we conducted two sensitivity analyses on the FAERS dataset: (1) restricting the analysis to reports where the target drug was listed as the sole suspected agent, after excluding all concomitant medications; and (2) specifically examining cases with documented improvement or resolution of the AEs following drug discontinuation, to assess the strength of dechallenge evidence.

### 2.4 Stratification analysis

We performed stratified signal detection and subgroup comparisons to evaluate age- and sex-related differences in ADRs associated with cetirizine and levocetirizine. These analyses focused on the top 20 most frequently reported PTs within each sex and age stratum: individuals under 18 years (pediatric population), those aged 18–64 years (adult population), and those aged ≥65 years (geriatric population).

### 2.5 Time to onset (TTO) analysis`

In this study, time to onset (TTO) of neuropsychiatric ADEs was defined as the interval between medication start date (THER.START_DT) and event date (DEMO.EVENT_DT). After excluding cases with missing data or ADEs occurring prior to medication initiation, Kaplan-Meier survival analysis was applied to compare the timing of neurologic and psychiatric ADEs between the cetirizine and levocetirizine.

Data extraction, cleaning, and statistical analysis were performed using SAS 9.4.

## 3 Results

### 3.1 Descriptive characteristics

**FIGURE 2:**
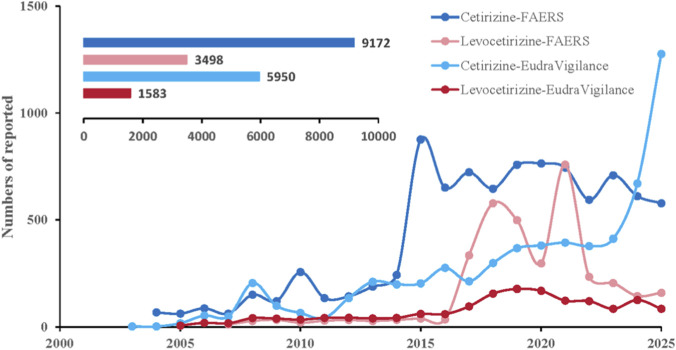
Time course of adverse neuropsychiatric events for cetirizine and levocetirizine.

**FIGURE 3:**
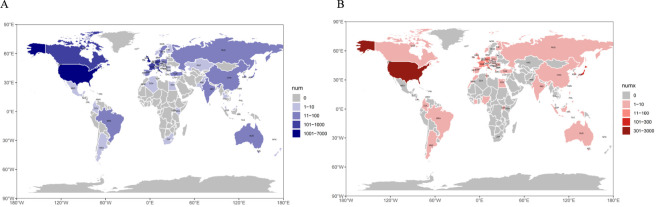
Geographic distribution of adverse neuropsychiatric event reports for cetirizine and levocetirizine (FAERS). **(A)** Cetirizine. **(B)** Levocetirizine. Darker shading corresponds to a greater number of reports per country.

FAERS (2004–2025) contained 9,172 neuropsychiatric ADEs for cetirizine (since 2004) and 3,498 for levocetirizine (since 2007), while EudraVigilance (2002–2025) included 5,950 cetirizine reports (since 2002) and 1,583 for levocetirizine (since 2005). Neuropsychiatric ADEs for both drugs showed a clear upward trend from 2015 onward in both databases (Figure 2; Table 1; Supplementary Table S2) summarize the clinical characteristics of these FAERS and EudraVigilance ADEs. Neuropsychiatric events showed significant gender and age disparities in both databases: reports were less frequent in males than in females and predominantly concentrated in the 18–65 age group. The majority of reports in FAERS originated from consumers, whereas in EudraVigilance most were submitted by healthcare professionals. Regarding clinical outcomes in FAERS, other serious outcomes were most common, followed by initial or prolonged hospitalization. Figure 3 depicts the national distribution in FAERS, showing the United States as the primary reporting country, though reports in EudraVigilance are predominantly from European Union countries.

**TABLE 1:** Characteristics of adverse neuropsychiatric event reports for cetirizine and levocetirizine in the FAERS database.

Characteristics	Cetirizine (N = 9172)	Levocetirizine (N = 3498)
Sex, n (%)		
Female	4788 (52.20)	1619 (46.28)
Male	2320 (25.29)	537 (15.35)
Unknow	2064 (22.50)	1342 (38.36)
Age (years), n (%)		
<18	1044 (11.38)	203 (5.80)
18–64	2780 (30.31)	522 (14.92)
≥65	1284 (14.00)	299 (8.55)
Unknow	4064 (44.31)	2474 (70.73)
Reporter category, n (%)		
Healthcare professional	2785 (26.66)	494 (14.12)
Consumer	6387 (69.64)	2954 (84.45)
Unknown	340 (3.71)	50 (1.43)
Outcome, n (%)^a^		
Hospitalization-initial or prolonged	1129 (12.31)	203 (5.80)
Disability	427 (4.66)	41 (1.17)
Life-Threatening	321 (3.50)	49 (1.40)
Death	217 (2.37)	49 (1.40)
Congenital anomaly	43 (0.47)	3 (0.09)
Required intervention	34 (0.37)	6 (0.17)
Other serious outcomes	3627 (39.54)	732 (20.93)
Unknown	4690 (51.13)	2596 (74.21)

^a^ Percentages are calculated based on the total number of reports for each drug (N). As a single report can be associated with more than one reporter category and/or outcome category, the sum of individual category counts may exceed N, and corresponding percentages are not mutually exclusive and may total >100%.

We also analyzed the indications and concomitant medications for cetirizine and levocetirizine in the FAERS database (Supplementary Tables S3, S4). After excluding reports classified as “Product used for unknown indication” and “Not specified,” the top five indications for both cetirizine and levocetirizine in FAERS were allergy-related conditions. The most common reported concomitant substance (per database records) for cetirizine was paracetamol, whereas for levocetirizine, the most common reported concomitant substance (per database records) was cetirizine (reflecting raw reporting data, not clinical concomitant medication).

### 3.2 Neuropsychiatric ADEs of cetirizine and levocetirizine

Supplementary Table S5 summarizes signal intensity and case counts for cetirizine and levocetirizine in two SOCs: nervous system disorders and psychiatric disorders. Both drugs exhibited significantly higher report numbers and signal metrics for nervous system disorders in both databases, with the exception of levocetirizine in EudraVigilance, where higher signals were observed for psychiatric disorders compared to nervous system disorders.

**FIGURE 4:**
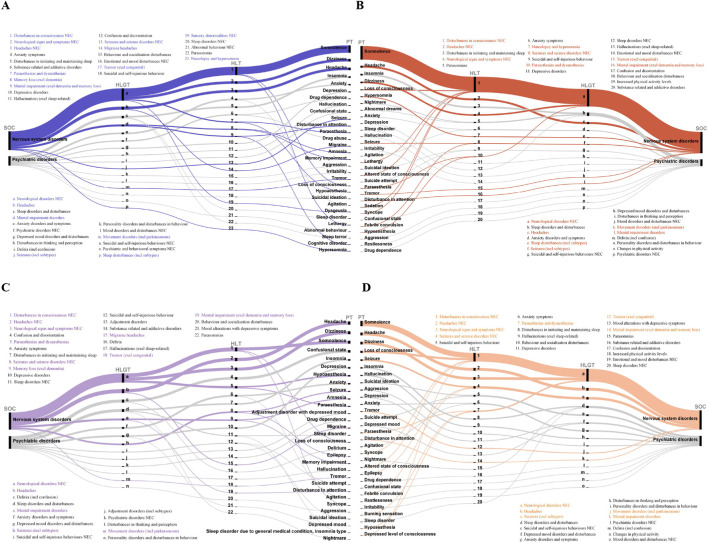
The top 30 reported neuropsychiatric PTs for cetirizine and levocetirizine, with their corresponding high-level terms (HLT), high-level group terms (HLGT), and system organ classes (SOC). **(A)** Cetirizine in FAERS. **(B)** Levocetirizine in FAERS. **(C)** Cetirizine in EudraVigilance. **(D)** Levocetirizine in EudraVigilance.

The top 30 PTs ranked by reported case counts and their corresponding High-Level Terms (HLT), High-Level Group Terms (HLGT), and SOCs for cetirizine- and levocetirizine-associated neuropsychiatric ADEs were systematically analyzed, alongside signal intensity and case counts (Figure 4; Table 2; Supplementary Tables S6–S8). The most prevalent HLT for both drugs was “Neurological disorders not elsewhere classified (NEC).” Aside from levocetirizine ranking third in sleep-related issues in the FAERS database, both drugs shared the same top three HLGTs: “Disturbances in consciousness NEC,” “Neurological signs and symptoms NEC,” and “Headaches NEC”.

**TABLE 2:** Disproportionality signals for the top 30 preferred terms (PTs) associated with cetirizine in the FAERS database.

PT	N	ROR (95% Cl)	PRR (χ2)	IC (IC025)	EBGM (EBGM05)
Somnolence	2946	10.14 (9.77,10.52)	9.85 (23,130.9)	3.28 (3.22)	9.71 (9.36)
Dizziness	1019	1.36 (1.28,1.45)	1.36 (96.41)	0.44 (0.35)	1.36 (1.28)
Headache	1003	1.06 (1.00,1.13)	1.06 (3.81)	0.09 (0.00)	1.06 (1.00)
Insomnia	658	1.63 (1.51,1.76)	1.63 (159.34)	0.70 (0.59)	1.63 (1.51)
Anxiety	496	1.15 (1.05,1.25)	1.14 (9.07)	0.19 (0.06)	1.14 (1.05)
Depression	349	1.00 (0.90,1.11)	1.00 (0.00)	0.00 (-0.15)	1.00 (0.90)
Drug dependence	281	1.11 (0.98,1.24)	1.11 (2.85)	0.14 (-0.03)	1.11 (0.98)
Hallucination	270	2.44 (2.16,2.75)	2.43 (227.35)	1.28 (1.10)	2.43 (2.15)
Confusional state	269	1.10 (0.98,1.24)	1.10 (2.51)	0.14 (-0.04)	1.10 (0.98)
Seizure	263	1.01 (0.89,1.14)	1.01 (0.02)	0.01 (-0.17)	1.01 (0.89)
Disturbance in attention	253	3.07 (2.71,3.47)	3.06 (350.40)	1.61 (1.42)	3.05 (2.70)
Paraesthesia	238	0.99 (0.87,1.13)	0.99 (0.01)	−0.01 (-0.20)	0.99 (0.87)
Drug abuse	226	1.79 (1.57,2.04)	1.78 (77.85)	0.83 (0.64)	1.78 (1.56)
Migraine	200	1.42 (1.23,1.63)	1.42 (24.60)	0.50 (0.30)	1.42 (1.23)
Amnesia	199	2.00 (1.74,2.30)	2.00 (99.01)	1.00 (0.78)	1.99 (1.74)
Memory impairment	195	0.93 (0.81,1.08)	0.93 (0.88)	−0.10 (-0.30)	0.94 (0.81)
Aggression	191	2.50 (2.17,2.88)	2.50 (170.94)	1.32 (1.10)	2.49 (2.16)
Irritability	189	2.06 (1.79,2.38)	2.06 (102.85)	1.04 (0.82)	2.06 (1.78)
Tremor	182	0.72 (0.62,0.83)	0.72 (19.83)	−0.47 (-0.68)	0.72 (0.62)
Loss of consciousness	180	0.93 (0.80,1.07)	0.93 (1.03)	−0.11 (-0.32)	0.93 (0.80)
Hypoaesthesia	178	0.78 (0.67,0.90)	0.78 (11.15)	−0.36 (-0.57)	0.78 (0.67)
Suicidal ideation	172	1.24 (1.07,1.44)	1.24 (7.99)	0.31 (0.09)	1.24 (1.07)
Agitation	170	1.51 (1.30,1.75)	1.51 (28.99)	0.59 (0.37)	1.51 (1.30)
Dysgeusia	166	1.45 (1.24,1.68)	1.44 (22.71)	0.53 (0.30)	1.44 (1.24)
Sleep disorder	160	1.55 (1.33,1.81)	1.55 (31.32)	0.63 (0.40)	1.55 (1.33)
Lethargy	157	1.80 (1.53,2.10)	1.79 (55.09)	0.84 (0.60)	1.79 (1.53)
Abnormal behaviour	150	2.45 (2.09,2.88)	2.45 (128.04)	1.29 (1.04)	2.44 (2.08)
Sleep terror	143	23.65 (20.01,27.95)	23.62 (2984.12)	4.51 (4.06)	22.79 (19.28)
Cognitive disorder	138	1.99 (1.68,2.35)	1.99 (67.56)	0.99 (0.73)	1.98 (1.68)
Hypersomnia	138	3.21 (2.71,3.79)	3.20 (208.31)	1.67 (1.41)	3.19 (2.70)

PTs-level mapping revealed that the two agents share an almost overlapping adverse-event profile, yet a handful of distinct signals remain. In the FAERS database, the five most frequently reported PTs for cetirizine were somnolence, dizziness, headache, insomnia, and anxiety; for levocetirizine, they were somnolence, headache, insomnia, dizziness and loss of consciousness. Although the EudraVigilance database largely reproduced this hierarchy, confusional state emerged among the top five PTs for cetirizine, and seizure for levocetirizine. Notably, with the sole exception of a marginally higher depression signal for levocetirizine in EudraVigilance, both databases consistently documented elevated anxiety and depression signals for cetirizine, even though the algorithmic assays remained negative. Among nearly all sleep-related PTs (e.g., insomnia, nightmare), cetirizine displayed significantly higher reporting rates and disproportionality scores than levocetirizine in the two databases. In both FAERS and EudraVigilance, amnesia and memory impairment emerged among cetirizine’s top 30 PTs with consistently elevated disproportionality, whereas levocetirizine exhibited no corresponding signal; furthermore, FAERS uniquely identified cognitive disorder for cetirizine. Importantly, migraine emerged only among cetirizine-linked PTs in the top-30 stratum.

### 3.3 Sensitivity analysis of the top 30 PTs for cetirizine and levocetirizine in FAERS

To ensure the robustness and reliability of the analyses, we conducted a sensitivity analysis of the top 30 PTs ranked by reported case counts for cetirizine and levocetirizine within the FAERS database (Table 3; Supplementary Tables S9–S11). The results showed that the top-five PTs for both agents still included somnolence, dizziness, and headache. Anxiety and depression again displayed higher disproportionality signals for cetirizine, whereas sleep-related events (insomnia, nightmare, etc.) continued to exhibit stronger signals for levocetirizine. Importantly, amnesia and memory impairment previously associated with cetirizine were excluded from the top-30 stratum under either analytical scenario. Moreover, migraine likewise dropped out of the top-30 PTs. Notably, both sensitivity analyses indicated higher signals for drug dependence associated with cetirizine.

**TABLE 3:** Significant signals on the top 30 PTs in cetirizine-monotherapy users.

PT	N	ROR (95% Cl)	PRR (χ2)	IC (IC025)	EBGM (EBGM05)
Somnolence	2030	15.88 (15.19,16.61)	15.16 (26,641.2)	3.91 (3.83)	15.01 (14.35)
Dizziness	474	1.42 (1.30,1.55)	1.42 (58.20)	0.50 (0.37)	1.42 (1.29)
Headache	395	0.94 (0.85,1.04)	0.94 (1.59)	−0.09 (-0.24)	0.94 (0.85)
Insomnia	286	1.59 (1.41,1.79)	1.59 (61.96)	0.66 (0.49)	1.58 (1.41)
Drug dependence	222	1.97 (1.72,2.24)	1.96 (104.55)	0.97 (0.77)	1.96 (1.72)
Anxiety	177	0.92 (0.79,1.06)	0.92 (1.38)	−0.13 (-0.34)	0.92 (0.79)
Depression	173	1.11 (0.96,1.29)	1.11 (1.94)	0.15 (-0.07)	1.11 (0.96)
Irritability	135	3.31 (2.79,3.92)	3.30 (216.05)	1.72 (1.45)	3.29 (2.78)
Seizure	120	1.03 (0.86,1.23)	1.03 (0.12)	0.04 (-0.22)	1.03 (0.86)
Abnormal behaviour	120	4.40 (3.68,5.27)	4.39 (313.58)	2.13 (1.83)	4.38 (3.66)
Dysgeusia	110	2.15 (1.78,2.59)	2.15 (67.40)	1.10 (0.81)	2.15 (1.78)
Aggression	110	3.23 (2.68,3.89)	3.22 (168.38)	1.69 (1.38)	3.22 (2.67)
Suicidal ideation	107	1.73 (1.43,2.09)	1.73 (32.98)	0.79 (0.50)	1.73 (1.43)
Hallucination	102	2.06 (1.70,2.50)	2.06 (55.48)	1.04 (0.74)	2.06 (1.69)
Paraesthesia	101	0.94 (0.78,1.15)	0.94 (0.33)	−0.08 (-0.37)	0.94 (0.78)
Hypersomnia	100	5.21 (4.28,6.35)	5.20 (338.56)	2.38 (2.03)	5.19 (4.26)
Anger	92	3.97 (3.23,4.87)	3.96 (203.30)	1.98 (1.64)	3.95 (3.22)
Confusional state	91	0.83 (0.68,1.03)	0.84 (2.96)	−0.26 (-0.56)	0.84 (0.68)
Nightmare	89	3.79 (3.07,4.66)	3.78 (181.52)	1.92 (1.57)	3.77 (3.06)
Lethargy	85	2.18 (1.76,2.70)	2.18 (54.08)	1.12 (0.79)	2.18 (1.76)
Tremor	83	0.74 (0.59,0.91)	0.74 (7.83)	−0.44 (-0.75)	0.74 (0.59)
Disturbance in attention	82	2.22 (1.79,2.76)	2.22 (55.05)	1.15 (0.81)	2.22 (1.79)
Burning sensation	75	1.59 (1.27,2.00)	1.59 (16.40)	0.67 (0.33)	1.59 (1.27)
Mood swings	73	3.37 (2.68,4.24)	3.36 (121.05)	1.75 (1.37)	3.36 (2.67)
Loss of consciousness	69	0.80 (0.63,1.01)	0.80 (3.58)	−0.33 (-0.67)	0.80 (0.63)
Psychomotor hyperactivity	69	6.08 (4.80,7.70)	6.07 (290.88)	2.60 (2.15)	6.05 (4.77)
Agitation	58	1.15 (0.89,1.49)	1.15 (1.17)	0.20 (-0.18)	1.15 (0.89)
Sleep disorder	52	1.13 (0.86,1.48)	1.13 (0.78)	0.18 (-0.22)	1.13 (0.86)
Depressed mood	50	1.43 (1.09,1.89)	1.43 (6.53)	0.52 (0.10)	1.43 (1.09)
Abnormal dreams	49	2.55 (1.93,3.38)	2.55 (46.20)	1.35 (0.90)	2.55 (1.93)

### 3.4 Subgroup analysis of neuropsychiatric ADEs associated with cetirizine and levocetirizine

**FIGURE 5:**
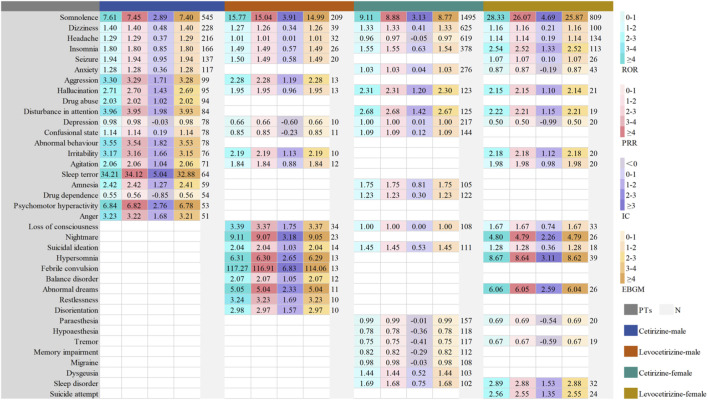
Subgroup analysis by sex in FAERS: Signal strength of the top 20 PTs for cetirizine and levocetirizine.

**FIGURE 6:**
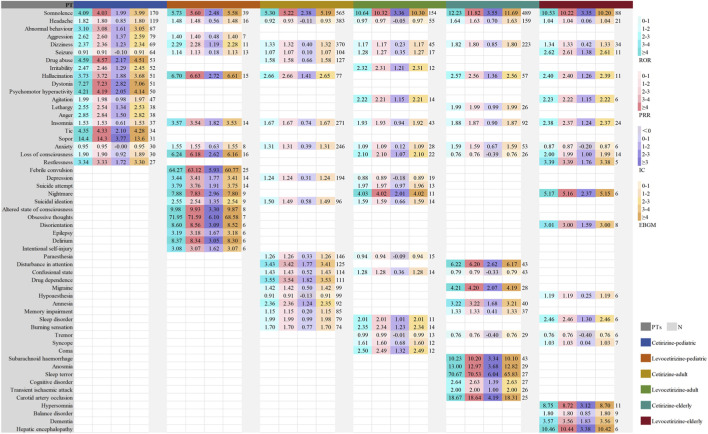
Subgroup analysis by age group in FAERS: Signal strength of the top 20 PTs for cetirizine and levocetirizine (pediatric: ≤18 years, adult: 19–64 years, and elderly: ≥65 years).

To assess neuropsychiatric ADEs of cetirizine and levocetirizine by age and sex (<18 years for pediatric; 18–64 years for adult; ≥65 years for elderly), stratified analyses were conducted. Using the top 20 reported PTs in each subgroup, we analyzed ADE distribution patterns and overlap between groups (Figures 5, 6; Supplementary Figures S2–S4). Across all pre-specified strata, disproportionality signals for somnolence, headache, dizziness, and insomnia were consistently detected for both cetirizine and levocetirizine. Consistent with the primary analysis, sleep-related ADEs (e.g., nightmares, abnormal dreams) and serious psychiatric events (e.g., suicidal ideation, attempts) among the top 20 PTs were predominantly associated with levocetirizine or demonstrated stronger signals than those for cetirizine across all sex subgroups. In males, mood-related events (e.g., anxiety, depression) and signals for drug dependence/abuse within the top 20 PTs were more frequently linked to cetirizine or exhibited higher signal strength compared to levocetirizine. Although migraine, epilepsy, delirium, dysgeusia, and memory impairment/amnesia did not reach statistical significance in some datasets, all were reported in females using cetirizine. Notably, febrile convulsion showed a strong signal in males treated with levocetirizine.

Cross-age group analysis identified somnolence as the most frequently reported ADE, with prominent signal strength.The top 20 PTs for cetirizine frequently included drug dependence, drug abuse, memory-related impairments (e.g., amnesia, memory impairment), and migraine, predominantly showing positive signals. In contrast, the top 20 PTs for levocetirizine were more concentrated on sleep-related disorders (e.g., nightmare, sleep disorder) and serious psychiatric events (e.g., suicide attempt, suicidal ideation), with generally stronger signal intensities. Notably, signal strengths for most ADEs were significantly lower in adults than in pediatric and elderly populations, suggesting that enhanced safety monitoring is warranted for pediatric and elderly patients. In pediatric patients, levocetirizine was associated with febrile convulsion and epilepsy within the top 20 PTs in both databases. Among elderly patients, both databases indicated a strong positive signal between cetirizine and subarachnoid hemorrhage. Furthermore, the FAERS data reported severe neurological events associated with cetirizine, including transient ischemic attack and carotid artery occlusion, while levocetirizine was specifically associated with hepatic encephalopathy.

### 3.5 Time-to-onset of neuropsychiatric events in FAERS

Kaplan–Meier survival curves (Supplementary Figure S5) illustrate the cumulative incidence of ADEs for cetirizine and levocetirizine by sex and age. Pooled sex- and age-stratified cohorts showed that neuropsychiatric adverse events for both drugs followed a uniform pattern: rapid accumulation within the first 30 days, plateauing by day 90. However, the timing of events was jointly modulated by drug type and demographic stratum. For cetirizine, the median cumulative event count was 0 (IQR: 0–6) in males and 0 (IQR: 0–11) in females, with a statistically significant sex-based difference in event timing (P = 0.0332). For levocetirizine, the median count was 1 (IQR: 0–11) in males and 1 (IQR: 0–16) in females, with no significant inter-sex divergence in timing (P = 0.7258). Across age groups, the median cumulative event counts for cetirizine were 0 (0–3) in the <18 years group, 1 (0–25) in the 18–64 years group, and 0 (0–2) in the ≥65 years group, showing distinct event timing across strata (P < 0.0001). The corresponding values for levocetirizine were 1 (0–10), 1 (0–9), and 3 (0.5–71.5), which also differed significantly by age (P = 0.0057).

## 4 Discussion

We conducted a systematic pharmacovigilance analysis of neuropsychiatric ADEs associated with cetirizine and levocetirizine using the FAERS and EudraVigilance databases. By evaluating data across the overall population and stratifying by sex and age, we provided quantitative evidence to support clinical risk-benefit assessments.

The most prominent ADE identified for both drugs was somnolence, a finding consistent with existing literature. A systematic review on the efficacy and safety of treatments for difficult-to-control chronic spontaneous urticaria noted that drowsiness is the main adverse reaction and that dose escalation of cetirizine may increase this risk (Iriarte Sotés et al., 2021). Another review on off-label high doses of second-generation antihistamines for spontaneous urticaria also cited drowsiness and headache as the main reactions for these drugs (Podder et al., 2023). A systematic review reported that levocetirizine carries a mild sedative risk (risk ratio 1.67), which is not significantly different from other second-generation antihistamines (Snidvongs et al., 2017). Furthermore, a randomized, double-blind, crossover, placebo-controlled trial in patients with perennial allergic rhinitis found that individuals with a history of mild to moderate sedation from cetirizine are unlikely to experience a different sedative response to levocetirizine (Tzanetos et al., 2011).

Disproportionality analyses conducted in this study consistently demonstrated that the signal strength for mood disorder-related adverse events (e.g., anxiety, depression) was predominantly lower with levocetirizine than with cetirizine across primary, sensitivity, and subgroup analyses. A potential mechanism involves the disruption of histamine metabolism. In the oxidative deamination metabolic pathway, histamine is converted to imidazoleacetic acid (IAA), which is then oxidized and ribosylated for transport. IAA-ribosylation may be involved in regulating synaptic transmission in the hippocampus, a crucial area of the brain for mood regulation (Bozdagi et al., 2011). As H1 receptor antagonists, cetirizine and levocetirizine may interfere with this process. Additionally, histamine promotes prostaglandin (PG) synthesis—particularly PGE2 and PGI2—via H1 receptors. Blocking H1 receptors may dysregulate PG production, which has been linked to neuropsychiatric symptoms, as PGE2 is known to reduce central dopamine levels (Van Kammen et al., 1989). Cetirizine’s racemic formulation may contribute to its higher neuropsychiatric risk. Only the R-enantiomer (levocetirizine) has significant H1 antagonistic activity. The S-enantiomer (dextrocetirizine) has much lower H1 affinity but may still interfere with neurotransmitter systems like GABA and 5-HT, potentially increasing the risk of mood disturbances (Tillement et al., 2003; Corsico et al., 2019). A controlled trial of 92 patients with chronic pruritus reported significantly higher rates of anxiety and depression in the cetirizine group than in the levocetirizine group (Ozdemir et al., 2014). Notably, while levocetirizine showed stronger disproportionality signals for serious psychiatric adverse events (e.g., suicide attempts, suicidal ideation) than cetirizine in primary and subgroup analyses, this trend reversed in the monotherapy sensitivity analysis. This discrepancy may be explained by concomitant medication use. Montelukast—a frequent co-prescribed drug with both cetirizine and levocetirizine—has itself been linked to neuropsychiatric effects such as mood changes and suicidal ideation (Sobczak and Pawliczak, 2025). Furthermore, since these antihistamines are mainly used for allergic diseases, and given that suicidal ideation/attempts represent a major fatal comorbidity in chronic spontaneous urticaria (CSU)—where second-generation H1-antihistamine monotherapy and omalizumab are associated with significantly lower mortality—the more common combination of levocetirizine with other antihistamines suggests possible suboptimal disease control, which could elevate the risk of serious psychiatric events (Kolkhir et al., 2025). Based on these findings, it remains unclear whether cetirizine or levocetirizine is more suitable for patients with psychiatric histories, and further studies are needed to clarify their comparative safety.

Our analysis revealed stronger pharmacovigilance signals for sleep-related ADEs with levocetirizine than with cetirizine. This may be explained by their effects on central histaminergic pathways. Histaminergic neurons, primarily located in the tuberomammillary nucleus (TMN) of the posterior hypothalamus, project broadly throughout the brain—including key sleep-regulating regions such as the ventrolateral preoptic nucleus (VLPO) (Brown et al., 2001). GABAergic neurons in these areas inhibit histaminergic activity by releasing GABA into the TMN, thereby reducing arousal and promoting sleep onset (Liu et al., 2010). Normally, histamine promotes wakefulness during the day, while decreased histamine activity at night facilitates sleep. As H1 receptor antagonists capable of crossing the blood–brain barrier in small amounts, both cetirizine and levocetirizine can disrupt this balance by impairing histaminergic signaling. Notably, levocetirizine has approximately twice the affinity for human H1 receptors compared to cetirizine, potentially leading to more pronounced effects on sleep architecture (Gillard et al., 2002). In some individuals, a drop in drug levels between doses—especially in the early morning hours—may cause rebound allergy symptoms or mild arousal, a phenomenon potentially more pronounced with levocetirizine. Although comparative studies are limited, both drugs have shown equivalent efficacy in improving sleep in patients with pruritus (Ozdemir et al., 2014). However, according to several studies, both can result in sleep difficulties. One study found an increased incidence of nightmares in pediatric patients when levocetirizine was used alongside montelukast (Altaş et al., 2023), while case reports have described exacerbation of pre-existing sleep terrors with cetirizine (Hussain and Aziz, 2021). Regarding insomnia, given that levocetirizine is frequently co-administered with other antihistamines, it is plausible that inadequate control of allergic pruritus in these patients contributes to sleep disturbances, thereby influencing the observed outcomes. In conclusion, patients at elevated risk of sleep disturbances may benefit more from cetirizine or alternative antihistamines with lower central nervous system activity.

Cetirizine is more frequently associated with reports of drug dependence and abuse than levocetirizine. Pharmacokinetic differences may underlie this observation. Levocetirizine exhibits a higher plasma protein binding rate (91.2% vs. 88%–90% for cetirizine), resulting in a significantly lower apparent volume of distribution (Bree et al., 2002). A lower volume of distribution allows therapeutic concentrations to be achieved with lower doses, thereby reducing interindividual variability in drug efficacy (Walsh et al., 2001). These pharmacokinetic advantages likely contribute to levocetirizine’s lower potential for dependence and abuse, supporting its consideration as a preferred option for long-term antihistamine therapy.

Elevated migraine signals associated with cetirizine were not sustained in the monotherapy sensitivity analysis, where migraine fell outside the top 30 PTs. This inconsistency may reflect confounding influences. Epidemiologic studies link migraine with allergic disorders, and histamine—via hypothalamic modulation—contributes to migraine pathophysiology and severity (Ferretti et al., 2023). Since cetirizine is primarily prescribed for allergic conditions, its use in patients with a higher baseline migraine risk may introduce confounding. Moreover, acetaminophen use frequently co-prescribed with cetirizine according to concomitant medication analysis, could further distort signal detection. Monotherapy analysis likely attenuated these confounders, yielding a more distinct signal profile. Similarly, after the monotherapy sensitivity analysis, cognition-related PTs did not rank within the top 30. Subsequent age subgroup analysis revealed that the elevated signals for cognitive impairment associated with cetirizine were primarily distributed among adult and elderly populations. This suggests that cognitive issues may themselves represent underlying comorbidities; to manage clinical symptoms, patients often require multiple concomitant medications beyond antihistamines alone, which likely explains the attenuation or disappearance of these signals in the monotherapy analysis. Nevertheless, existing studies still indicate that cetirizine can exert some degree of influence on cognitive function (van Ruitenbeek et al., 2010). However, studies have shown that levocetirizine did not demonstrate interference with objective measures of cognitive function after both single and repeated doses, and similarly, cetirizine showed no evidence of cognitive impairment after either acute or repeated administration (Hindmarch et al., 2001).

A population-based analysis revealed that neuropsychiatric ADEs related to cetirizine and levocetirizine were more frequently reported in women than in men. Gender-related physiological differences significantly influence drug pharmacokinetics (Lee et al., 2024). Women typically absorb drugs more slowly due to lower gastric acid secretion, delayed gastric emptying—especially during mid-menstruation—and reduced gastrointestinal blood flow. During pregnancy, gastric acid secretion decreases by 40%, while intestinal motility increases by 30%–50%, further altering absorption profiles (Fletcher et al., 1994). Additionally, women have a higher body fat percentage, lower total and plasma volumes, and fluctuating sex hormone levels, all of which affect drug distribution. Both cetirizine and levocetirizine are primarily eliminated via the kidneys. Since men generally have higher serum and urinary creatinine levels and greater creatinine clearance, they may excrete these drugs more efficiently than women. Lastly, consumer reports make up the majority of ADE data in FAERS, and sociocultural factors may drive higher reporting rates among women and adults (Lee et al., 2023).

Across gender subgroups, the pattern of differences between cetirizine and levocetirizine was generally consistent with the primary analysis, with the notable exception of febrile convulsions, which were specifically reported in males in association with levocetirizine. The observed differences between cetirizine and levocetirizine across genders may stem from sex-based distinctions in neural circuit function, hormone-receptor interactions, epigenetic regulation, evolutionary adaptations, and pathophysiological responses (Bangasser and Cuarenta, 2021). Additionally, this may reflect sex-specific modulation of the central histaminergic system, a key regulator of neural excitability (Kim et al., 2023). Cetirizine and levocetirizine may interfere with the central histaminergic neuronal system, causing epilepsy and tremors. Histamine lowers striatal dopamine levels in male mice, but increases striatal dopamine levels in some female mice, and this sex difference in neurotransmitter modulation provides an important foundation for understanding the neurological ADEs of antihistamine drugs (Van Zandt and Pittenger, 2025). Although the molecular mechanisms underlying these sex differences remain unclear and the findings may be influenced by imbalanced sample sizes, these observations still provide valuable insights for implementing sex-differentiated clinical practice.

Age-stratified analyses revealed weaker disproportionality signals in adults despite higher absolute reporting frequencies—a pattern warranting longitudinal surveillance due to potential data reporting artifacts. Special attention should be paid to pediatric and elderly populations due to their distinct physiology. In pediatric patients, levocetirizine demonstrated stronger pharmacovigilance signals for febrile convulsions and epilepsy—conditions with an elevated baseline prevalence in children (Gilsoul et al., 2019; Novorol et al., 2007; AlDaithan et al., 2024). While limited studies directly compare the safety profiles of cetirizine and levocetirizine in pediatric populations, existing evidence suggests that cetirizine is generally well-tolerated and does not significantly elevate systemic side effect risks beyond somnolence (Zhou et al., 2022). Hence, cetirizine is preferred over levocetirizine in pediatric patients. In the elderly, cetirizine was associated with severe organic neurological disorders, including subarachnoid hemorrhage—a finding of particular importance given its high fatality rate and poor prognosis (Tan et al., 2024) —as well as transient ischemic attack and carotid artery occlusion. Given that cognitive decline is a prominent feature of geriatric disorders, our finding that cetirizine was associated with reports of cognitive impairment warrants attention. Additionally, levocetirizine was linked to a higher risk of hepatic encephalopathy, a significant contributor to hospitalization, readmission, and death in patients with cirrhosis; the 1-year mortality rate for severe hepatic encephalopathy can be as high as 42% (Sahney and Wadhawan, 2022). These findings emphasize the need for vigilance regarding drug-induced subarachnoid hemorrhage, particularly with cetirizine in older adults, and suggest that levocetirizine should be avoided in patients with cirrhosis. Notably, as many adverse events may relate to age-related comorbidities not analyzed here, further studies are required to confirm these observations.

According to the TTO analysis, neuropsychiatric adverse events for both cetirizine and levocetirizine accumulated rapidly within 0–30 days post-initiation and plateaued by day 90, indicating that intensive early-phase monitoring, rather than long-term follow-up, may be more efficient for signal detection. Sex-stratified comparisons revealed a statistically significant difference in event-timing for cetirizine (P < 0.05) but not for levocetirizine, consistent with the more stable pharmacokinetics of the latter after structural optimization. In the elderly, cetirizine exhibited a “compressed” distribution (IQR 0.00–2.00 days), whereas levocetirizine showed a “dispersed-delayed” pattern (IQR 0.00–71.00 days); increased blood–brain barrier permeability and drug–drug interactions likely underlie this divergence. Therefore, the active surveillance window for levocetirizine in older adults should be extended to ≥90 days. Among adults aged 18–64 years, cetirizine demonstrated the greatest temporal dispersion (IQR 0.00–25.00 days), warranting individualized and prolonged follow-up in this population.

Although previous studies utilizing the FAERS database have investigated the neurological ADEs of antihistamines such as cetirizine and levocetirizine, they did not comprehensively examine psychiatric ADEs or provide detailed evaluations of gender and age differences (Hu et al., 2024). The current study systematically analyzed both drugs’ neuropsychiatric ADE profiles and, through gender-age stratified analyses, first revealed their population-specific safety differences, providing a comprehensive clinical medication guide. However, several limitations remain. In-depth analysis was impeded by concerns with underreporting and inadequate information in the FAERS database. Due to database deficiencies, it was also impossible to directly assess the relative risk differences between the two medicines. Additionally, the lack of detailed patient clinical information, including comorbidities, impeded the adjustment for potential confounders. Moreover, disproportionality analyses can only suggest potential signals and do not establish causality or measure true risk. The true incidence of ADEs could not be determined due to the absence of prescription population data. Given the high rate of missing data for key covariates such as age and gender in the FAERS database, performing multivariate regression would substantially reduce the sample size and introduce unreliable statistical assumptions. Therefore, multivariate logistic regression was considered not feasible in this study. Consequently, strong signals detected may reflect high reporting rates rather than true incidence, potentially biasing safety assessments. These limitations underscore the need for cautious interpretation of the findings and further research to validate the results.

## 5 Conclusion

This study employed pharmacovigilance analyses based on the FAERS and EudraVigilance databases to assess the neuropsychiatric safety profiles of cetirizine and levocetirizine. Somnolence, dizziness, headache, and insomnia were common ADEs with high signal strengths for both drugs, representing key shared central nervous system risks that require close attention in clinical practice. Cetirizine generally exhibited higher signal intensities for depression, anxiety, and drug abuse/dependence than levocetirizine, with unique associations with cognitive impairment and migraine. In contrast, levocetirizine showed a more concentrated signal for sleep-related ADEs, and its use warrants vigilance for serious psychiatric events such as suicide attempts and suicidal ideation. Subgroup analysis revealed population heterogeneity. Among levocetirizine users, febrile convulsions were more frequently reported in males, while pediatric patients presented with febrile convulsions and epilepsy. In the elderly, cetirizine was associated with subarachnoid hemorrhage, transient ischemic attack, and carotid artery occlusion, whereas levocetirizine was linked to hepatic encephalopathy. These findings indicate the necessity for individualized risk assessment in elderly patients based on their comorbidities.

In summary, this study has systematically identified distinct neuropsychiatric safety profiles for cetirizine and levocetirizine, thereby providing a crucial foundation for rational therapeutic decision-making.

## Data Availability

The original contributions presented in the study are included in the article/Supplementary Material, further inquiries can be directed to the corresponding author.
