# Supplementary material for: Gender and age variations in neuropsychiatric adverse events of cetirizine and levocetirizine: a disproportionality analysis of ICSRs from FAERS and EudraVigilance data
Source: Front Pharmacol. 2026 Jan 9;16:1737359. doi: 10.3389/fphar.2025.1737359 (PMC12827542; doi:10.3389/fphar.2025.1737359)
Supplement: Supplementary file 1 [file Supplementaryfile1.docx]

1. **Methods**
   1. **Data sources**

The raw data for this study were sourced from two publicly accessible pharmacovigilance databases. Data from the U.S. FDA Adverse Event Reporting System (FAERS) were obtained in ASCII format from the official FDA website (<https://fis.fda.gov/extensions/FPD-QDE-FAERS/FPD-QDE-FAERS.html>). The FAERS database, publicly available since 2004 Q1, is updated quarterly. Data from the European EudraVigilance database were downloaded at the Stakeholder Group (SG II) access level as Line Listing files from the official portal (<https://www.adrreports.eu/en/search_subst.html>). The EudraVigilance Line Listing data are updated weekly; data for this analysis were downloaded on November 14, 2025.

***The FDA Adverse Event Reporting System (FAERS)***

FAERS is a key pharmacovigilance database for post-marketing safety surveillance, containing adverse event reports from healthcare professionals, consumers, and manufacturers[1]. Its structure comprises seven datasets: DEMO (demographics), DRUG (drug information), REAC (reactions), OUTC (outcomes), RPSR (report sources), THER (therapy), and INDI (indications). We performed a retrospective disproportionality analysis using FAERS data (2004–2025) to investigate neuropsychiatric adverse events associated with cetirizine and levocetirizine, with a focus on age- and sex-specific differences.

***EudraVigilance***

EudraVigilance is a data and information management system developed by the European Medicines Agency (EMA) for collecting, managing, and analyzing reports of suspected side effects from medicines authorized or under clinical trial in the European Economic Area (EEA)[2]. As individual case safety reports (ICSRs) can be submitted by various parties (e.g., healthcare professionals, pharmaceutical industry, or patients), the system implements specific procedures to minimize duplicate entries[3]. Details regarding the data quality of EudraVigilance are provided on its official website (<https://www.adrreports.eu/en/data_quality.html>). Furthermore, all reported events are coded using the Medical Dictionary for Regulatory Activities (MedDRA), in compliance with regulatory requirements[2].

- 1. **Disproportionality analysis**

**Table S1.** Methods, formulas, and thresholds for Reporting Odds Ratio (ROR), Proportional Reporting Ratio (PRR), Multi-Item Gamma Poisson Shrinker (MGPS), and Bayesian Confidence Propagation Neural Network (BCPNN).

| Algorithms | Equation | Criteria |
| --- | --- | --- |
| ROR | ROR=ad/bc  95%CI =eln (ROR) ± 1.96(1/a + 1/b + 1/c + 1/d) ^0.5 | lower limit of 95% CI > 1, N ≥ 3 |
| PRR | PRR = a (c + d)/ c (a + b)  χ2= [(ad – bc) ^2] (a + b + c + d)/ [(a + b) (c + d) (a + c) (b + d)] IC = log2a (a + b + c + d)/ ((a + c) (a + b) | N ≥ 3 PRR ≥ 2, χ2 ≥ 4, N ≥ 3 |
| MGPS | 95%CI = e ln (EBGM) ± 1.96(1/a + 1/b + 1/c + 1/d) ^0.5 | EBGM05 > 2 |
| BCPNN | 95%CI = E(IC) ± 2[V(IC)] ^0.5  EBGM = a (a + b + c + d)/ (a + c)/ (a + b) | IC025 > 0 |

1. Count of reports featuring both the specified drug and target adverse events; (b) number of reports involving other adverse drug events alongside the specified drug; (c) reports with the target adverse events but without the specified drug; (d) reports without the specified drug and without the target adverse events. EBGM: Empirical Bayesian Geometric Mean; 95% CI: 95% confidence interval; N: number of reports; χ2: chi-squared; IC: information component; IC025: lower limit of 95% CI of the IC; E (IC): IC expectations; V(IC): variance of IC; EBGM05: lower limit of 95% CI of EBGM.

**1.3 The flow chart of EudraVigilance**


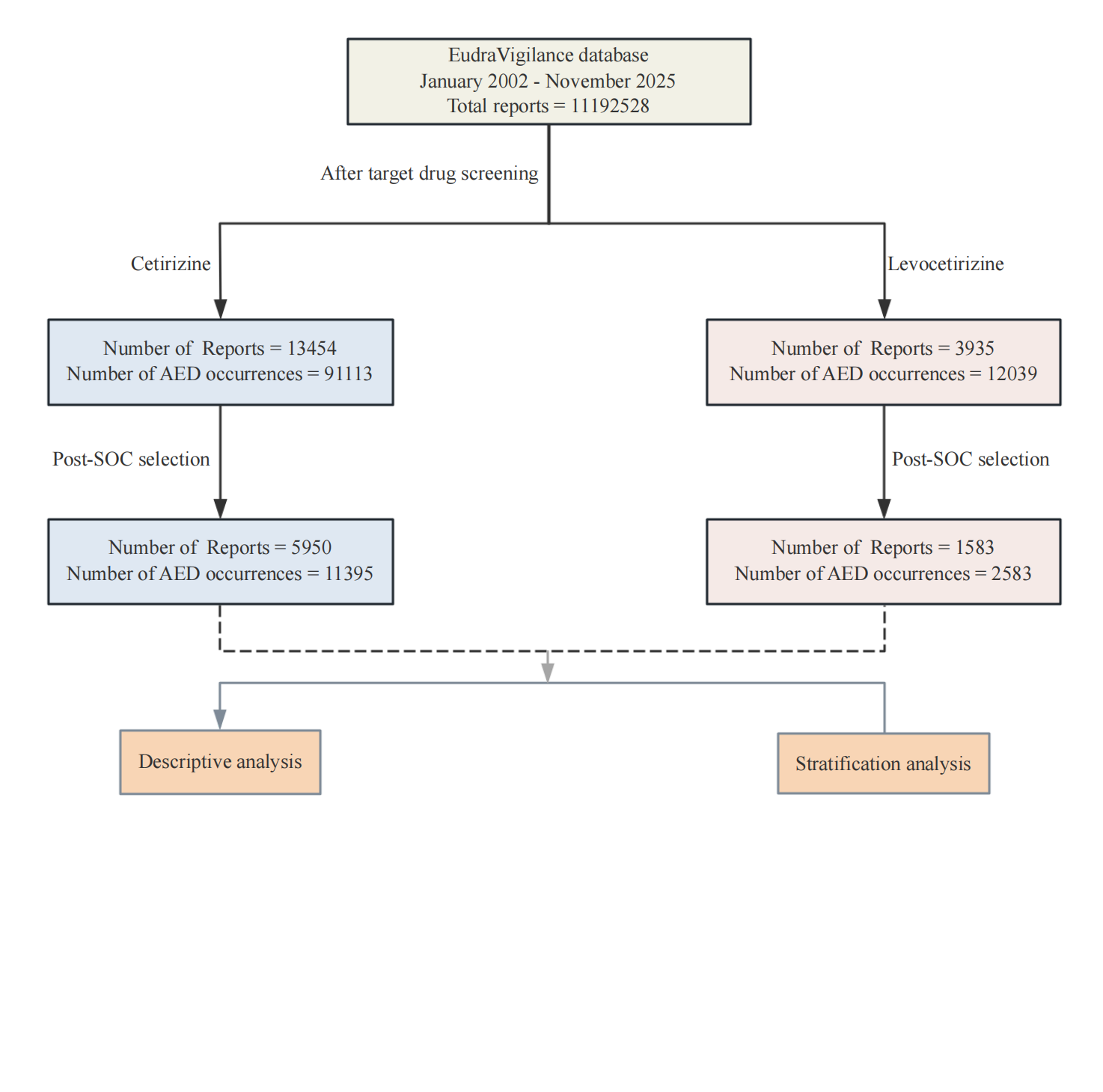


**Figure S1.** The flow chart of the study of EudraVigilance.

1. **Result**

**2.1 Descriptive characteristics**

**Table S2.** Characteristics of adverse neuropsychiatric event reports for cetirizine and levocetirizine in the EudraVigilance database.

|  | **Cetirizine (N=5950)** | **Levocetirizine (N=1583)** |
| --- | --- | --- |
| **Sex, n (%)** |  |  |
| Female | 4202(70.62) | 899(56.79) |
| Male | 1403(23.58) | 493(31.14) |
| Unknow | 345(5.80) | 191(12.07) |
| **Age (years), n (%)** |  |  |
| <18 | 695(11.68) | 246(15.54) |
| 18-64 | 2988(50.22) | 667(42.14) |
| ≥65 | 668(11.23) | 228(14.40) |
| Unknow | 1599(26.87) | 442(27.92) |
| **Reported person, n (%)** |  |  |
| Healthcare Professional (%) | 3854(64.77) | 816(51.55) |
| Non-Healthcare Professional (%) | 1987(33.39) | 764(48.26) |
| Not Specified (%) | 109(1.83) | 3(0.19) |
| **Reported country, n (%)** |  |  |
| European Economic Area (%) | 2409(40.49) | 820(51.80) |
| Non-European Economic Area (%) | 3541(59.51) | 763(48.20) |

**Table S3.** Top five indications for cetirizine and levocetirizine in FAERS after excluding reports with "product used for unknown indication" and "not specified" categories.

| Cetirizine | | Levocetirizine | |
| --- | --- | --- | --- |
| Indication | N (%) | Indication | N (%) |
| Hypersensitivity | 919(10.02) | Hypersensitivity | 616(17.61) |
| Seasonal allergy | 809(8.82) | Seasonal allergy | 293(8.38) |
| Multiple allergies | 485(5.29) | Multiple allergies | 163(4.66) |
| Pruritus | 204(2.22) | Rhinitis allergic | 76(2.17) |
| Rhinitis allergic | 174(1.90) | Urticaria | 49(1.40) |

**Table S4.** Top ten concomitant medications of cetirizine and levocetirizine in FAERS after excluding reports with "VARIOUS" categories.

| Top ten concomitant medications of Cetirizine | | Top ten concomitant medications of Levocetirizine | |
| --- | --- | --- | --- |
| Drug | N | Drug | N |
| PARACETAMOL | 529 | CETIRIZINE | 94 |
| LEVOTHYROXINE | 361 | LORATADINE | 81 |
| MONTELUKAST | 283 | PARACETAMOL | 71 |
| SALBUTAMOL | 266 | DIPHENHYDRAMINE | 67 |
| ACETYLSALICYLIC ACID | 264 | FEXOFENADINE | 67 |
| COLECALCIFEROL | 245 | MONTELUKAST | 53 |
| IBUPROFEN | 241 | ACETYLSALICYLIC ACID | 50 |
| SIMVASTATIN | 233 | FLUTICASONE | 48 |
| FLUTICASONE | 226 | COLECALCIFEROL | 39 |
| LANSOPRAZOLE | 221 | LEVOTHYROXINE | 34 |

**2.2 Neuropsychiatric ADEs associated with cetirizine and levocetirizine**

**Table S5.** Signal intensity of neuropsychiatric ADEs concerning cetirizine and levocetirizine at the SOC level.

|  |  | Fears | | EudraVigilance | |
| --- | --- | --- | --- | --- | --- |
|  |  | Nervous system disorders | Psychiatric disorders | Nervous system disorders | Psychiatric disorders |
| Cetirizine | N | 10351 | 6416 | 6522 | 4873 |
|  | ROR (95%Cl) | 1.36(1.33,1.39) | 1.25(1.22,1.29) | 0.73(0.71,0.75) | 1.03(1.01,1.06) |
|  | PRR (χ^2^) | 1.32(863.48) | 1.24(306.06) | 0.75(589.37) | 1.03(5.30) |
|  | EBGM (EBGM05) | 1.32(1.29) | 1.24(1.20) | 0.75(0.73) | 1.03(1.00) |
|  | IC (IC025) | 0.40(0.37) | 0.31(0.27) | -0.41(-0.45) | 0.05(0.00) |
| levocetirizine | N | 3345 | 1347 | 1654 | 929 |
|  | ROR (95%Cl) | 2.20(2.12,2.29) | 1.24(1.17,1.31) | 1.51(1.44,1.60) | 1.53(1.43,1.64) |
|  | PRR (χ^2^) | 2.00(1829.54) | 1.22(56.53) | 1.44(249.38) | 1.49(157.82) |
|  | EBGM (EBGM05) | 2.00(1.93) | 1.22(1.15) | 1.44(1.37) | 1.49(1.39) |
|  | IC (IC025) | 1.00(0.95) | 0.29(0.21) | 0.53(0.45) | 0.58(0.48) |

**Table S6.** Disproportionality signals for the top 30 PTs associated with levocetirizine in the FAERS database.

| PT | N | ROR (95%Cl) | PRR (χ^2^) | IC (IC025) | EBGM (EBGM05) |
| --- | --- | --- | --- | --- | --- |
| Somnolence | 1777 | 30.42(28.97,31.95) | 27.79(45612.8) | 4.78(4.69) | 27.54(26.22) |
| Headache | 256 | 1.28(1.13,1.45) | 1.28(15.43) | 0.35(0.17) | 1.28(1.13) |
| Insomnia | 219 | 2.56(2.24,2.93) | 2.55(206.15) | 1.35(1.14) | 2.54(2.23) |
| Dizziness | 204 | 1.28(1.11,1.47) | 1.28(12.35) | 0.35(0.15) | 1.28(1.11) |
| Loss of consciousness | 116 | 2.82(2.35,3.39) | 2.81(135.52) | 1.49(1.20) | 2.81(2.34) |
| Hypersomnia | 93 | 10.19(8.31,12.50) | 10.15(764.77) | 3.34(2.91) | 10.12(8.25) |
| Nightmare | 90 | 8.05(6.54,9.90) | 8.02(551.50) | 3.00(2.59) | 8.00(6.50) |
| Anxiety | 72 | 0.78(0.62,0.98) | 0.78(4.45) | -0.36(-0.69) | 0.78(0.62) |
| Abnormal dreams | 72 | 7.90(6.26,9.96) | 7.87(431.06) | 2.97(2.51) | 7.85(6.23) |
| Depression | 60 | 0.81(0.63,1.04) | 0.81(2.74) | -0.31(-0.67) | 0.81(0.63) |
| Sleep disorder | 57 | 2.60(2.01,3.38) | 2.60(56.03) | 1.38(0.96) | 2.60(2.00) |
| Hallucination | 53 | 2.24(1.71,2.94) | 2.24(36.47) | 1.16(0.74) | 2.24(1.71) |
| Seizure | 50 | 0.90(0.68,1.19) | 0.90(0.54) | -0.15(-0.55) | 0.90(0.68) |
| Irritability | 45 | 2.31(1.72,3.09) | 2.30(33.19) | 1.20(0.74) | 2.30(1.72) |
| Agitation | 44 | 1.84(1.37,2.47) | 1.83(16.67) | 0.87(0.42) | 1.83(1.36) |
| Lethargy | 40 | 2.15(1.58,2.93) | 2.15(24.53) | 1.10(0.61) | 2.15(1.57) |
| Suicidal ideation | 39 | 1.32(0.97,1.81) | 1.32(3.06) | 0.40(-0.07) | 1.32(0.97) |
| Altered state of consciousness | 35 | 5.16(3.71,7.20) | 5.16(117.09) | 2.36(1.72) | 5.15(3.69) |
| Paraesthesia | 33 | 0.65(0.46,0.91) | 0.65(6.36) | -0.63(-1.11) | 0.65(0.46) |
| Tremor | 33 | 0.61(0.44,0.86) | 0.61(8.02) | -0.70(-1.18) | 0.61(0.44) |
| Suicide attempt | 33 | 1.69(1.20,2.38) | 1.69(9.23) | 0.75(0.23) | 1.69(1.20) |
| Disturbance in attention | 32 | 1.82(1.29,2.57) | 1.82(11.75) | 0.86(0.32) | 1.82(1.28) |
| Sedation | 31 | 3.85(2.70,5.47) | 3.84(65.12) | 1.94(1.31) | 3.84(2.70) |
| Syncope | 29 | 0.89(0.62,1.28) | 0.89(0.39) | -0.17(-0.69) | 0.89(0.62) |
| Confusional state | 29 | 0.56(0.39,0.80) | 0.56(10.17) | -0.84(-1.35) | 0.56(0.39) |
| Febrile convulsion | 28 | 74.85(51.42,108.94) | 74.74(1986.58) | 6.19(3.85) | 72.91(50.09) |
| Hypoaesthesia | 27 | 0.56(0.38,0.81) | 0.56(9.60) | -0.85(-1.37) | 0.56(0.38) |
| Aggression | 27 | 1.66(1.14,2.42) | 1.66(7.02) | 0.73(0.15) | 1.66(1.14) |
| Restlessness | 26 | 2.20(1.49,3.23) | 2.19(16.90) | 1.13(0.51) | 2.19(1.49) |
| Drug dependence | 25 | 0.46(0.31,0.68) | 0.46(15.66) | -1.11(-1.65) | 0.46(0.31) |

**Table S7.** Disproportionality signals for the top 30 PTs associated with cetirizine in the EudraVigilance database.

| PT | N | ROR (95%Cl) | PRR (χ^2^) | IC (IC025) | EBGM (EBGM05) |
| --- | --- | --- | --- | --- | --- |
| Headache | 898 | 0.72(0.67,0.77) | 0.72(99.04) | -0.47(-0.57) | 0.72(0.67) |
| Dizziness | 828 | 1.02(0.95,1.09) | 1.02(0.32) | 0.03(-0.07) | 1.02(0.95) |
| Somnolence | 808 | 2.34(2.18,2.51) | 2.33(612.85) | 1.22(1.11) | 2.32(2.17) |
| Confusional state | 679 | 2.36(2.18,2.54) | 2.35(523.48) | 1.23(1.11) | 2.34(2.17) |
| Insomnia | 438 | 1.36(1.24,1.49) | 1.36(41.42) | 0.44(0.30) | 1.36(1.24) |
| Depression | 403 | 1.35(1.23,1.49) | 1.35(36.52) | 0.43(0.29) | 1.35(1.22) |
| Hypoaesthesia | 398 | 1.64(1.49,1.81) | 1.64(99.51) | 0.71(0.57) | 1.64(1.48) |
| Anxiety | 389 | 1.06(0.96,1.17) | 1.06(1.16) | 0.08(-0.07) | 1.06(0.96) |
| Seizure | 276 | 0.89(0.79,1.00) | 0.89(3.77) | -0.17(-0.34) | 0.89(0.79) |
| Amnesia | 270 | 2.79(2.48,3.15) | 2.79(307.89) | 1.47(1.29) | 2.78(2.46) |
| Paraesthesia | 233 | 0.81(0.72,0.93) | 0.81(9.82) | -0.29(-0.48) | 0.82(0.72) |
| Adjustment disorder with depressed mood | 193 | 9.10(7.89,10.49) | 9.08(1364.60) | 3.16(2.89) | 8.94(7.76) |
| Drug dependence | 191 | 1.22(1.06,1.41) | 1.22(7.62) | 0.29(0.08) | 1.22(1.06) |
| Migraine | 188 | 1.52(1.31,1.75) | 1.51(32.83) | 0.60(0.38) | 1.51(1.31) |
| Sleep disorder | 182 | 1.53(1.32,1.77) | 1.53(32.95) | 0.61(0.39) | 1.52(1.32) |
| Loss of consciousness | 162 | 0.72(0.62,0.84) | 0.72(17.58) | -0.47(-0.70) | 0.72(0.62) |
| Delirium | 161 | 2.14(1.84,2.50) | 2.14(97.67) | 1.10(0.86) | 2.14(1.83) |
| Epilepsy | 159 | 2.11(1.81,2.47) | 2.11(92.30) | 1.07(0.83) | 2.10(1.80) |
| Memory impairment | 158 | 1.26(1.08,1.47) | 1.26(8.52) | 0.33(0.10) | 1.26(1.08) |
| Hallucination | 158 | 1.42(1.22,1.66) | 1.42(19.59) | 0.50(0.27) | 1.42(1.21) |
| Tremor | 125 | 0.50(0.42,0.60) | 0.50(61.91) | -0.99(-1.24) | 0.50(0.42) |
| Suicide attempt | 118 | 0.81(0.68,0.98) | 0.82(4.95) | -0.29(-0.56) | 0.82(0.68) |
| Disturbance in attention | 115 | 1.20(1.00,1.44) | 1.20(3.71) | 0.26(-0.01) | 1.20(1.00) |
| Agitation | 113 | 0.95(0.79,1.14) | 0.95(0.36) | -0.08(-0.35) | 0.95(0.79) |
| Syncope | 112 | 0.49(0.41,0.59) | 0.49(58.47) | -1.02(-1.28) | 0.49(0.41) |
| Aggression | 109 | 1.48(1.22,1.78) | 1.48(16.74) | 0.56(0.28) | 1.48(1.22) |
| Suicidal ideation | 101 | 0.78(0.64,0.95) | 0.78(6.35) | -0.36(-0.64) | 0.78(0.64) |
| Depressed mood | 87 | 1.13(0.92,1.40) | 1.13(1.32) | 0.18(-0.13) | 1.13(0.92) |
| Nightmare | 86 | 1.76(1.43,2.18) | 1.76(28.21) | 0.81(0.49) | 1.76(1.42) |
| Sleep disorder due to general medical condition, insomnia type | 86 | 5.36(4.33,6.62) | 5.35(301.30) | 2.41(2.03) | 5.31(4.29) |

**Table S8.** Disproportionality signals for the top 30 PTs associated with levocetirizine in the EudraVigilance database

| PT | N | ROR (95%Cl) | PRR (χ^2^) | IC (IC025) | EBGM (EBGM05) |
| --- | --- | --- | --- | --- | --- |
| Somnolence | 336 | 7.51(6.74,8.37) | 7.33(1839.00) | 2.87(2.68) | 7.31(6.56) |
| Headache | 179 | 1.09(0.94,1.26) | 1.09(1.25) | 0.12(-0.10) | 1.09(0.94) |
| Dizziness | 168 | 1.57(1.35,1.83) | 1.57(34.67) | 0.65(0.42) | 1.57(1.34) |
| Loss of consciousness | 131 | 4.45(3.75,5.29) | 4.42(346.63) | 2.14(1.85) | 4.41(3.71) |
| Seizure | 99 | 2.43(1.99,2.96) | 2.42(82.52) | 1.27(0.96) | 2.42(1.98) |
| Insomnia | 82 | 1.93(1.55,2.40) | 1.92(36.47) | 0.94(0.61) | 1.92(1.55) |
| Hallucination | 69 | 4.72(3.72,5.97) | 4.69(200.58) | 2.23(1.81) | 4.69(3.70) |
| Suicidal ideation | 56 | 3.28(2.52,4.27) | 3.27(88.34) | 1.71(1.27) | 3.27(2.51) |
| Aggression | 51 | 5.25(3.99,6.91) | 5.23(174.46) | 2.39(1.87) | 5.23(3.97) |
| Anxiety | 50 | 1.03(0.78,1.36) | 1.03(0.04) | 0.04(-0.37) | 1.03(0.78) |
| Depression | 50 | 1.27(0.96,1.67) | 1.27(2.81) | 0.34(-0.07) | 1.27(0.96) |
| Tremor | 48 | 1.46(1.10,1.94) | 1.46(6.98) | 0.55(0.12) | 1.46(1.10) |
| Suicide attempt | 46 | 2.41(1.81,3.22) | 2.41(37.86) | 1.27(0.80) | 2.41(1.80) |
| Depressed mood | 42 | 4.15(3.06,5.61) | 4.14(99.82) | 2.05(1.50) | 4.13(3.05) |
| Paraesthesia | 41 | 1.09(0.80,1.48) | 1.09(0.28) | 0.12(-0.33) | 1.09(0.80) |
| Disturbance in attention | 38 | 3.00(2.18,4.12) | 2.99(50.44) | 1.58(1.05) | 2.99(2.18) |
| Agitation | 36 | 2.28(1.65,3.17) | 2.28(25.89) | 1.19(0.66) | 2.28(1.64) |
| Syncope | 34 | 1.13(0.81,1.59) | 1.13(0.54) | 0.18(-0.31) | 1.13(0.81) |
| Nightmare | 34 | 5.28(3.77,7.39) | 5.27(117.46) | 2.40(1.74) | 5.26(3.76) |
| Altered state of consciousness | 31 | 3.67(2.58,5.22) | 3.66(60.04) | 1.87(1.25) | 3.66(2.57) |
| Epilepsy | 28 | 2.81(1.94,4.07) | 2.80(32.53) | 1.49(0.86) | 2.80(1.93) |
| Drug dependence | 28 | 1.35(0.93,1.96) | 1.35(2.59) | 0.44(-0.12) | 1.35(0.93) |
| Confusional state | 27 | 0.70(0.48,1.03) | 0.70(3.37) | -0.51(-1.04) | 0.70(0.48) |
| Febrile convulsion | 26 | 6.46(4.39,9.49) | 6.45(119.50) | 2.69(1.87) | 6.44(4.38) |
| Restlessness | 24 | 2.13(1.43,3.18) | 2.13(14.37) | 1.09(0.45) | 2.13(1.43) |
| Irritability | 22 | 1.84(1.21,2.80) | 1.84(8.42) | 0.88(0.22) | 1.84(1.21) |
| Burning sensation | 21 | 2.14(1.40,3.29) | 2.14(12.79) | 1.10(0.41) | 2.14(1.40) |
| Sleep disorder | 21 | 1.33(0.87,2.04) | 1.33(1.73) | 0.41(-0.22) | 1.33(0.87) |
| Hypoaesthesia | 20 | 0.62(0.40,0.97) | 0.62(4.57) | -0.68(-1.29) | 0.62(0.40) |
| Depressed level of consciousness | 19 | 1.75(1.12,2.75) | 1.75(6.14) | 0.81(0.11) | 1.75(1.12) |

**2.3 Sensitivity analysis of the top 30 PTs for cetirizine and levocetirizine in FAERS**

**Table S9.** Top 30 dechallenge–confirmed PT Signals for Cetirizine.

| PT | N | ROR (95%Cl) | PRR (χ^2^) | IC (IC025) | EBGM (EBGM05) |
| --- | --- | --- | --- | --- | --- |
| Somnolence | 446 | 11.60(10.55,12.75) | 11.21(4152.96) | 3.48(3.31) | 11.19(10.18) |
| Dizziness | 220 | 2.25(1.97,2.57) | 2.23(150.36) | 1.16(0.95) | 2.23(1.95) |
| Headache | 195 | 1.58(1.37,1.82) | 1.57(41.17) | 0.65(0.44) | 1.57(1.37) |
| Depression | 122 | 2.68(2.24,3.20) | 2.66(126.91) | 1.41(1.13) | 2.66(2.23) |
| Anxiety | 115 | 2.03(1.69,2.44) | 2.02(59.46) | 1.01(0.73) | 2.02(1.68) |
| Insomnia | 96 | 1.81(1.48,2.21) | 1.81(34.64) | 0.85(0.55) | 1.81(1.48) |
| Aggression | 70 | 6.99(5.53,8.85) | 6.96(356.95) | 2.80(2.34) | 6.95(5.49) |
| Confusional state | 69 | 2.16(1.70,2.73) | 2.15(42.57) | 1.10(0.73) | 2.15(1.70) |
| Suicidal ideation | 66 | 3.64(2.85,4.63) | 3.62(125.39) | 1.86(1.45) | 3.62(2.84) |
| Disturbance in attention | 64 | 5.91(4.62,7.56) | 5.89(259.53) | 2.56(2.09) | 5.88(4.60) |
| Seizure | 58 | 1.70(1.31,2.19) | 1.69(16.47) | 0.76(0.37) | 1.69(1.31) |
| Lethargy | 58 | 5.06(3.91,6.55) | 5.04(187.98) | 2.33(1.86) | 5.04(3.89) |
| Loss of consciousness | 54 | 2.12(1.62,2.77) | 2.12(31.91) | 1.08(0.66) | 2.12(1.62) |
| Irritability | 51 | 4.24(3.22,5.58) | 4.23(125.65) | 2.08(1.59) | 4.22(3.21) |
| Anger | 50 | 7.33(5.55,9.68) | 7.30(271.77) | 2.87(2.29) | 7.29(5.52) |
| Hallucination | 46 | 3.16(2.36,4.22) | 3.15(67.53) | 1.65(1.17) | 3.15(2.36) |
| Disorientation | 44 | 5.52(4.11,7.42) | 5.50(162.09) | 2.46(1.89) | 5.50(4.09) |
| Tremor | 43 | 1.30(0.96,1.75) | 1.30(2.91) | 0.37(-0.07) | 1.30(0.96) |
| Paraesthesia | 43 | 1.37(1.01,1.84) | 1.37(4.22) | 0.45(0.00) | 1.37(1.01) |
| Abnormal behaviour | 43 | 5.35(3.96,7.22) | 5.33(151.35) | 2.41(1.84) | 5.33(3.95) |
| Hypersomnia | 42 | 7.43(5.49,10.06) | 7.41(232.52) | 2.89(2.25) | 7.40(5.46) |
| Agitation | 42 | 2.84(2.10,3.84) | 2.83(49.85) | 1.50(1.00) | 2.83(2.09) |
| Mood swings | 39 | 6.11(4.46,8.37) | 6.10(166.04) | 2.61(1.98) | 6.09(4.45) |
| Depressed mood | 34 | 3.31(2.37,4.64) | 3.31(54.73) | 1.72(1.14) | 3.31(2.36) |
| Sleep terror | 34 | 41.71(29.74,58.49) | 41.60(1335.47) | 5.37(3.77) | 41.24(29.41) |
| Syncope | 32 | 1.59(1.13,2.25) | 1.59(7.03) | 0.67(0.14) | 1.59(1.12) |
| Balance disorder | 32 | 1.86(1.31,2.63) | 1.86(12.66) | 0.89(0.35) | 1.86(1.31) |
| Paranoia | 30 | 8.72(6.09,12.49) | 8.70(204.25) | 3.12(2.28) | 8.69(6.07) |
| Epilepsy | 30 | 5.08(3.55,7.26) | 5.07(97.85) | 2.34(1.64) | 5.06(3.54) |
| Drug dependence | 29 | 0.87(0.60,1.25) | 0.87(0.58) | -0.20(-0.72) | 0.87(0.60) |

**Table S10.** Significant signals on the top 30 PTs in levocetirizine-monotherapy users.

| PT | N | ROR (95%Cl) | PRR (χ^2^) | IC (IC025) | EBGM (EBGM05) |
| --- | --- | --- | --- | --- | --- |
| Somnolence | 1493 | 40.89(38.73,43.17) | 36.23(50909.1) | 5.17(5.06) | 35.95(34.06) |
| Headache | 182 | 1.42(1.22,1.64) | 1.41(21.87) | 0.50(0.28) | 1.41(1.22) |
| Insomnia | 175 | 3.19(2.75,3.70) | 3.16(259.31) | 1.66(1.42) | 3.16(2.72) |
| Dizziness | 142 | 1.39(1.17,1.63) | 1.38(15.05) | 0.47(0.22) | 1.38(1.17) |
| Hypersomnia | 82 | 13.98(11.25,17.38) | 13.90(979.24) | 3.79(3.27) | 13.86(11.15) |
| Nightmare | 77 | 10.71(8.56,13.41) | 10.65(672.36) | 3.41(2.91) | 10.63(8.50) |
| Loss of consciousness | 72 | 2.72(2.16,3.43) | 2.71(77.84) | 1.44(1.07) | 2.71(2.15) |
| Abnormal dreams | 62 | 10.58(8.24,13.58) | 10.53(533.86) | 3.39(2.83) | 10.51(8.19) |
| Anxiety | 47 | 0.79(0.59,1.05) | 0.79(2.58) | -0.34(-0.75) | 0.79(0.59) |
| Sleep disorder | 44 | 3.12(2.32,4.20) | 3.12(63.24) | 1.64(1.14) | 3.11(2.32) |
| Depression | 43 | 0.90(0.67,1.21) | 0.90(0.48) | -0.15(-0.58) | 0.90(0.67) |
| Irritability | 37 | 2.95(2.13,4.07) | 2.94(47.46) | 1.56(1.02) | 2.94(2.13) |
| Lethargy | 35 | 2.92(2.10,4.08) | 2.92(44.19) | 1.55(0.99) | 2.92(2.09) |
| Hallucination | 31 | 2.04(1.43,2.90) | 2.04(16.37) | 1.03(0.47) | 2.04(1.43) |
| Agitation | 31 | 2.01(1.41,2.86) | 2.01(15.67) | 1.00(0.45) | 2.01(1.41) |
| Seizure | 25 | 0.70(0.47,1.04) | 0.70(3.22) | -0.51(-1.06) | 0.70(0.47) |
| Suicidal ideation | 24 | 1.26(0.85,1.89) | 1.26(1.32) | 0.34(-0.26) | 1.26(0.85) |
| Drug dependence | 23 | 0.66(0.44,0.99) | 0.66(4.01) | -0.60(-1.17) | 0.66(0.44) |
| Sedation | 22 | 4.24(2.79,6.44) | 4.24(54.35) | 2.08(1.29) | 4.23(2.79) |
| Aggression | 22 | 2.10(1.38,3.19) | 2.10(12.63) | 1.07(0.40) | 2.10(1.38) |
| Disturbance in attention | 22 | 1.94(1.28,2.95) | 1.94(10.03) | 0.96(0.30) | 1.94(1.28) |
| Paraesthesia | 20 | 0.61(0.39,0.94) | 0.61(5.02) | -0.71(-1.32) | 0.61(0.39) |
| Migraine | 20 | 1.04(0.67,1.61) | 1.04(0.02) | 0.05(-0.58) | 1.04(0.67) |
| Anger | 20 | 2.80(1.81,4.35) | 2.80(23.17) | 1.49(0.74) | 2.80(1.81) |
| Tremor | 19 | 0.55(0.35,0.86) | 0.55(7.04) | -0.86(-1.48) | 0.55(0.35) |
| Poor quality sleep | 16 | 3.66(2.24,5.98) | 3.66(30.87) | 1.87(0.96) | 3.65(2.24) |
| Restlessness | 15 | 1.97(1.19,3.27) | 1.97(7.13) | 0.98(0.17) | 1.97(1.18) |
| Memory impairment | 15 | 0.52(0.32,0.87) | 0.53(6.44) | -0.93(-1.61) | 0.53(0.32) |
| Altered state of consciousness | 15 | 3.43(2.07,5.70) | 3.43(25.82) | 1.78(0.85) | 3.43(2.07) |
| Confusional state | 14 | 0.42(0.25,0.71) | 0.42(11.33) | -1.26(-1.94) | 0.42(0.25) |

**Table S11.** Top 30 dechallenge–confirmed PT Signals for levocetirizine-monotherapy users.

| PT | N | ROR (95%Cl) | PRR (χ2) | IC (IC025) | EBGM (EBGM05) |
| --- | --- | --- | --- | --- | --- |
| Somnolence | 95 | 9.94(8.10,12.19) | 9.66(739.31) | 3.27(2.85) | 9.65(7.87) |
| Headache | 51 | 1.68(1.27,2.21) | 1.67(13.73) | 0.74(0.31) | 1.67(1.26) |
| Dizziness | 29 | 1.19(0.83,1.72) | 1.19(0.89) | 0.25(-0.29) | 1.19(0.83) |
| Depression | 27 | 2.40(1.64,3.50) | 2.38(21.78) | 1.25(0.64) | 2.38(1.63) |
| Insomnia | 24 | 1.83(1.23,2.74) | 1.83(9.04) | 0.87(0.24) | 1.83(1.22) |
| Suicidal ideation | 21 | 4.69(3.05,7.21) | 4.67(60.56) | 2.22(1.38) | 4.67(3.04) |
| Loss of consciousness | 20 | 3.19(2.06,4.95) | 3.18(29.88) | 1.67(0.89) | 3.18(2.05) |
| Anxiety | 20 | 1.43(0.92,2.21) | 1.42(2.52) | 0.51(-0.15) | 1.42(0.92) |
| Seizure | 19 | 2.25(1.43,3.54) | 2.25(13.15) | 1.17(0.43) | 2.24(1.43) |
| Syncope | 17 | 3.44(2.13,5.54) | 3.42(29.20) | 1.78(0.91) | 3.42(2.12) |
| Agitation | 15 | 4.11(2.48,6.83) | 4.10(35.15) | 2.03(1.06) | 4.10(2.47) |
| Nightmare | 15 | 8.78(5.29,14.59) | 8.74(102.86) | 3.13(1.83) | 8.74(5.26) |
| Febrile convulsion | 13 | 225.48(130.36,390.01) | 224.52(2859.45) | 7.79(2.95) | 221.94(128.31) |
| Hallucination | 13 | 3.62(2.10,6.23) | 3.60(24.48) | 1.85(0.83) | 3.60(2.09) |
| Depressed mood | 11 | 4.34(2.40,7.85) | 4.33(28.20) | 2.11(0.93) | 4.33(2.40) |
| Irritability | 11 | 3.70(2.05,6.69) | 3.69(21.59) | 1.88(0.76) | 3.69(2.04) |
| Confusional state | 11 | 1.39(0.77,2.51) | 1.39(1.20) | 0.47(-0.41) | 1.39(0.77) |
| Paraesthesia | 10 | 1.29(0.69,2.39) | 1.29(0.64) | 0.36(-0.55) | 1.29(0.69) |
| Abnormal dreams | 9 | 6.46(3.36,12.42) | 6.44(41.35) | 2.69(1.15) | 6.44(3.35) |
| Tremor | 9 | 1.10(0.57,2.11) | 1.10(0.08) | 0.14(-0.79) | 1.10(0.57) |
| Aggression | 8 | 3.22(1.61,6.45) | 3.22(12.23) | 1.69(0.40) | 3.22(1.61) |
| Disorientation | 8 | 4.06(2.03,8.12) | 4.05(18.38) | 2.02(0.63) | 4.05(2.02) |
| Cognitive disorder | 7 | 3.11(1.48,6.53) | 3.10(9.99) | 1.63(0.28) | 3.10(1.48) |
| Restlessness | 7 | 3.88(1.85,8.14) | 3.87(14.92) | 1.95(0.49) | 3.87(1.84) |
| Obsessive thoughts | 7 | 78.50(37.33,165.05) | 78.32(532.18) | 6.29(1.85) | 78.01(37.10) |
| Hypersomnia | 6 | 4.29(1.92,9.55) | 4.28(15.09) | 2.10(0.45) | 4.28(1.92) |
| Dementia | 6 | 4.50(2.02,10.02) | 4.49(16.29) | 2.17(0.49) | 4.49(2.02) |
| Depressed level of consciousness | 6 | 3.12(1.40,6.96) | 3.12(8.64) | 1.64(0.17) | 3.12(1.40) |
| Emotional distress | 6 | 0.99(0.44,2.20) | 0.99(0.00) | -0.02(-1.11) | 0.99(0.44) |
| Disturbance in attention | 6 | 2.24(1.00,4.98) | 2.23(4.09) | 1.16(-0.17) | 2.23(1.00) |

**2.4** **Subgroup analysis of neuropsychiatric ADEs associated with cetirizine and levocetirizine**


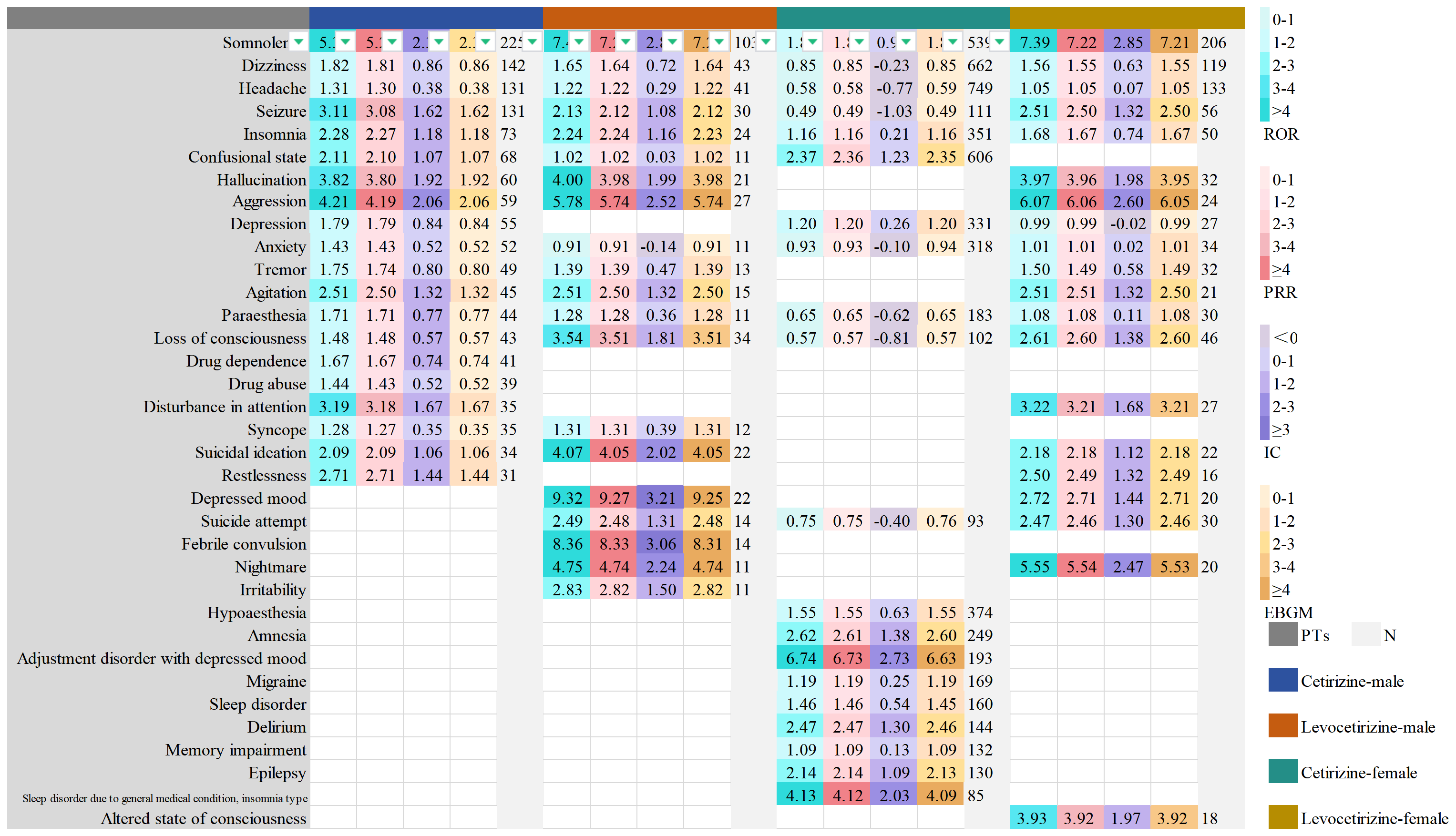


**Figure S2.** Subgroup analysis by sex in EudraVigilance: Signal strength of the top 20 PTs for cetirizine and levocetirizine.


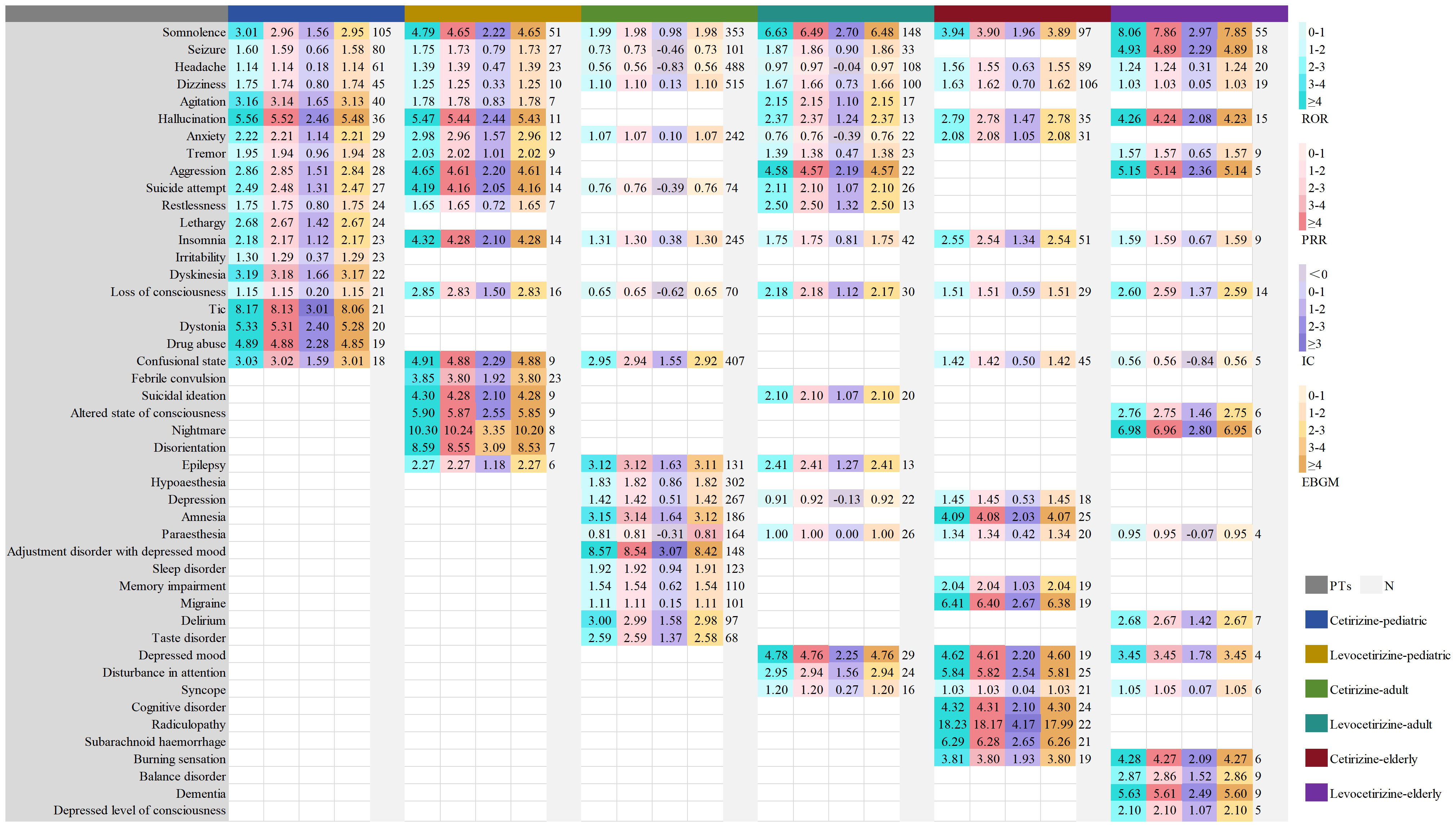


**Figure S3.** Subgroup analysis by age group in EudraVigilance: Signal strength of the top 20 PTs for cetirizine and levocetirizine (pediatric: ≤18 years, adult: 19–64 years, and elderly: ≥65 years).

.

A B


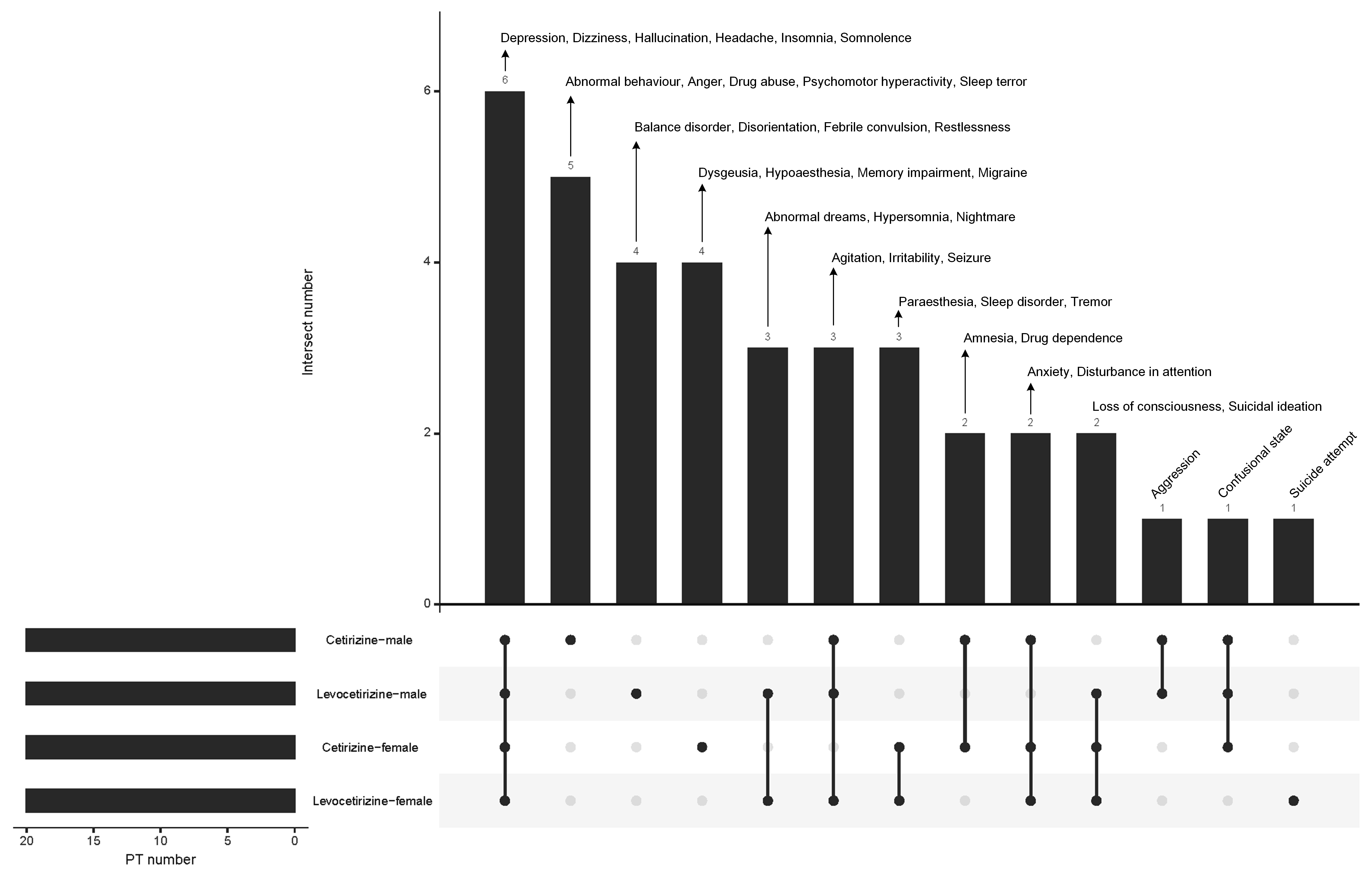

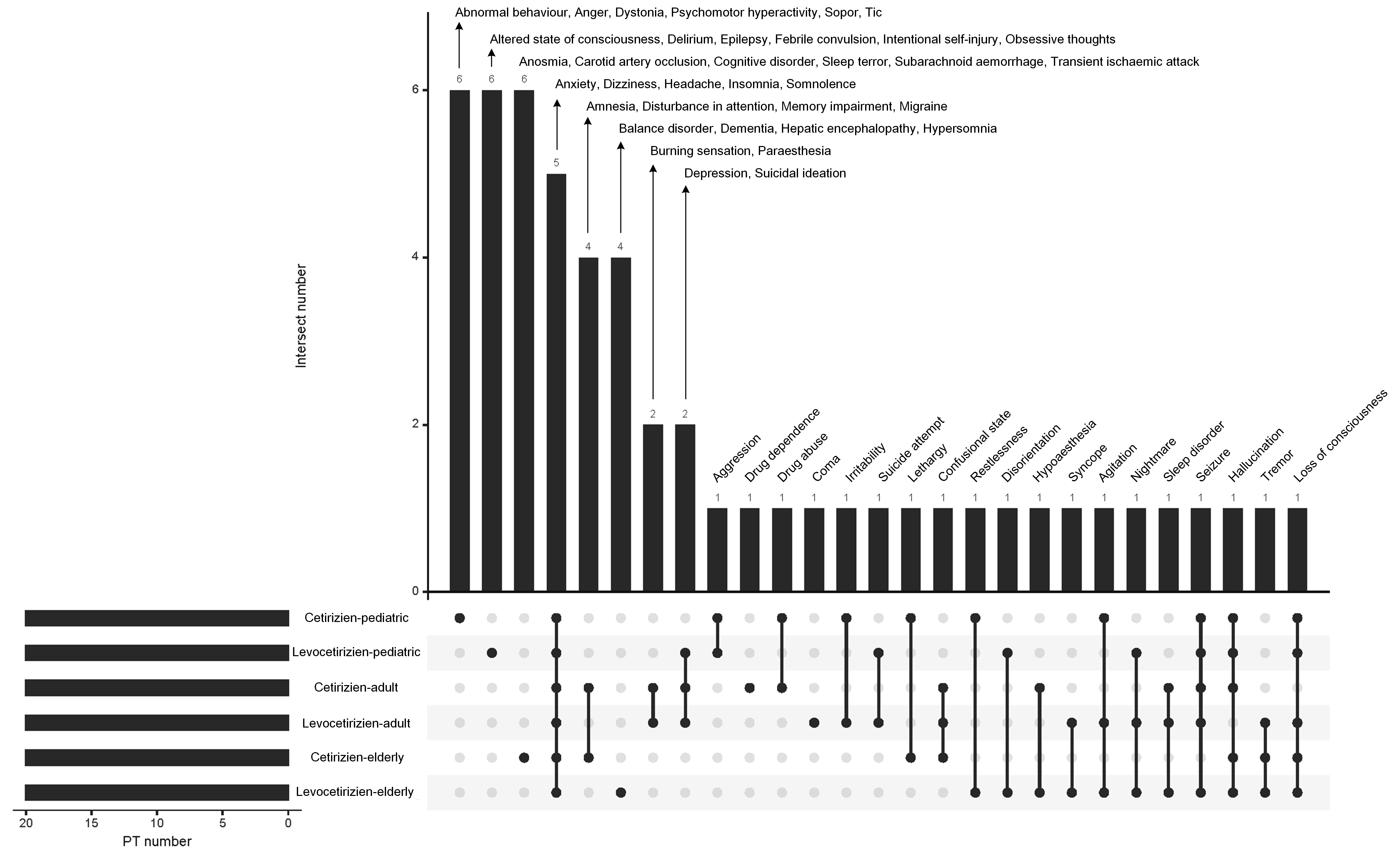


C D


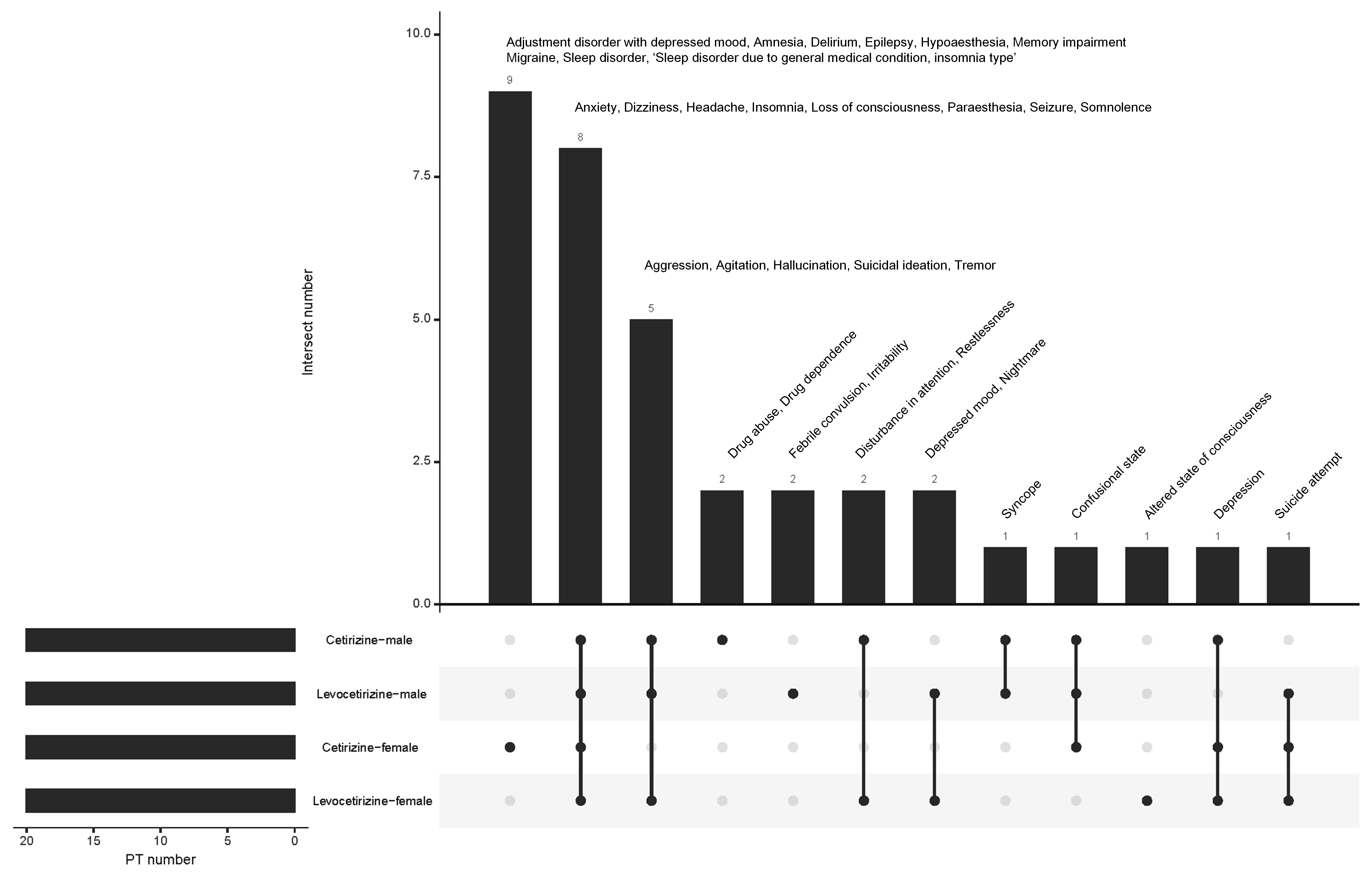

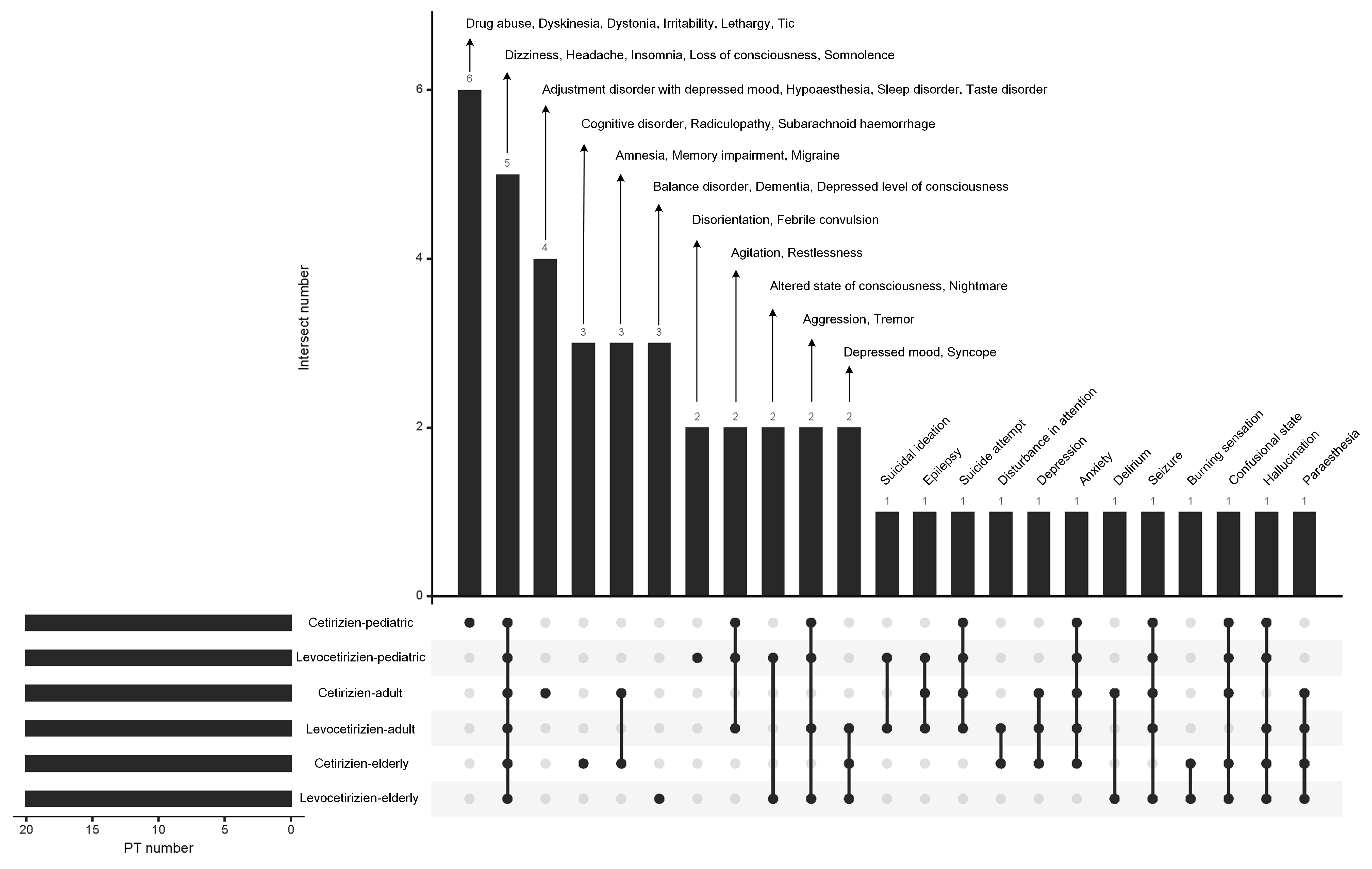


**Figure S4.** Intersection analysis of frequently reported neuropsychiatric adverse events across subpopulations. (A) Cetirizine & levocetirizine by sex (FAERS). (B) Cetirizine & levocetirizine by age (FAERS). (C) Cetirizine & levocetirizine by sex (EudraVigilance). (D) Cetirizine & levocetirizine by age (EudraVigilance). (The upset plots visualize intersections among the top 20 PTs by report count within each subgroup.)

**2.5** **Time-to-onset of neuropsychiatric events in FAERS.**

**A B**


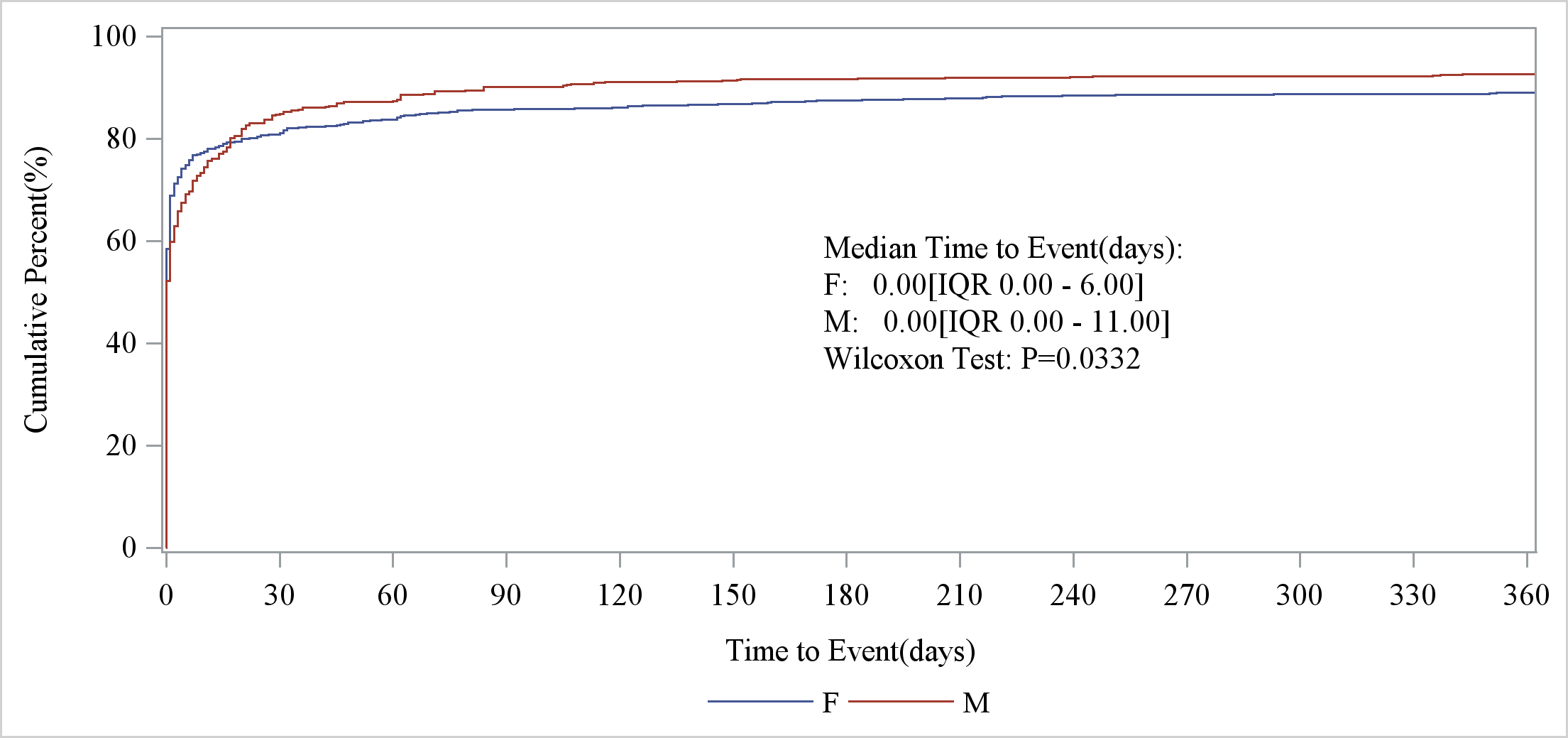

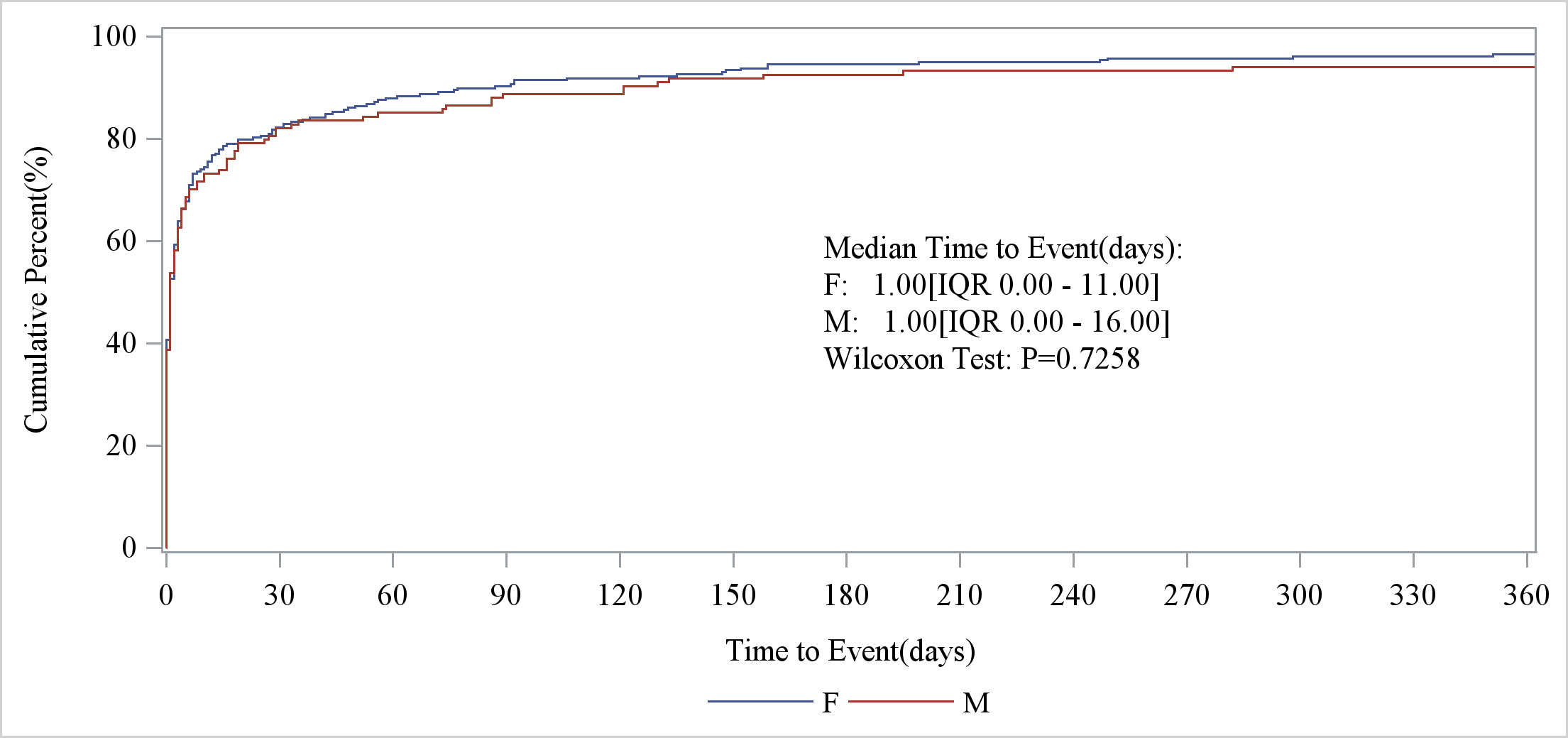


**C D**


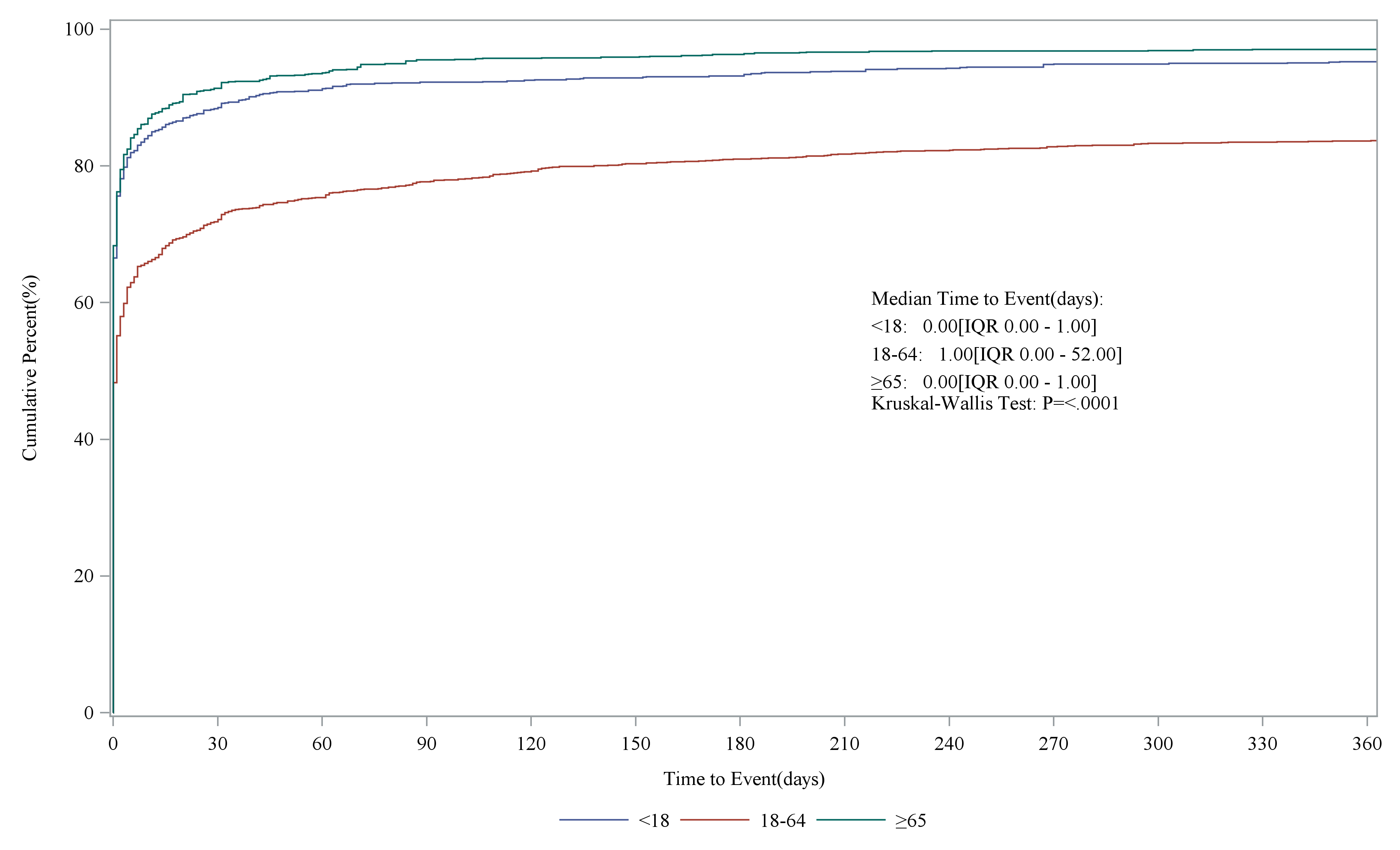

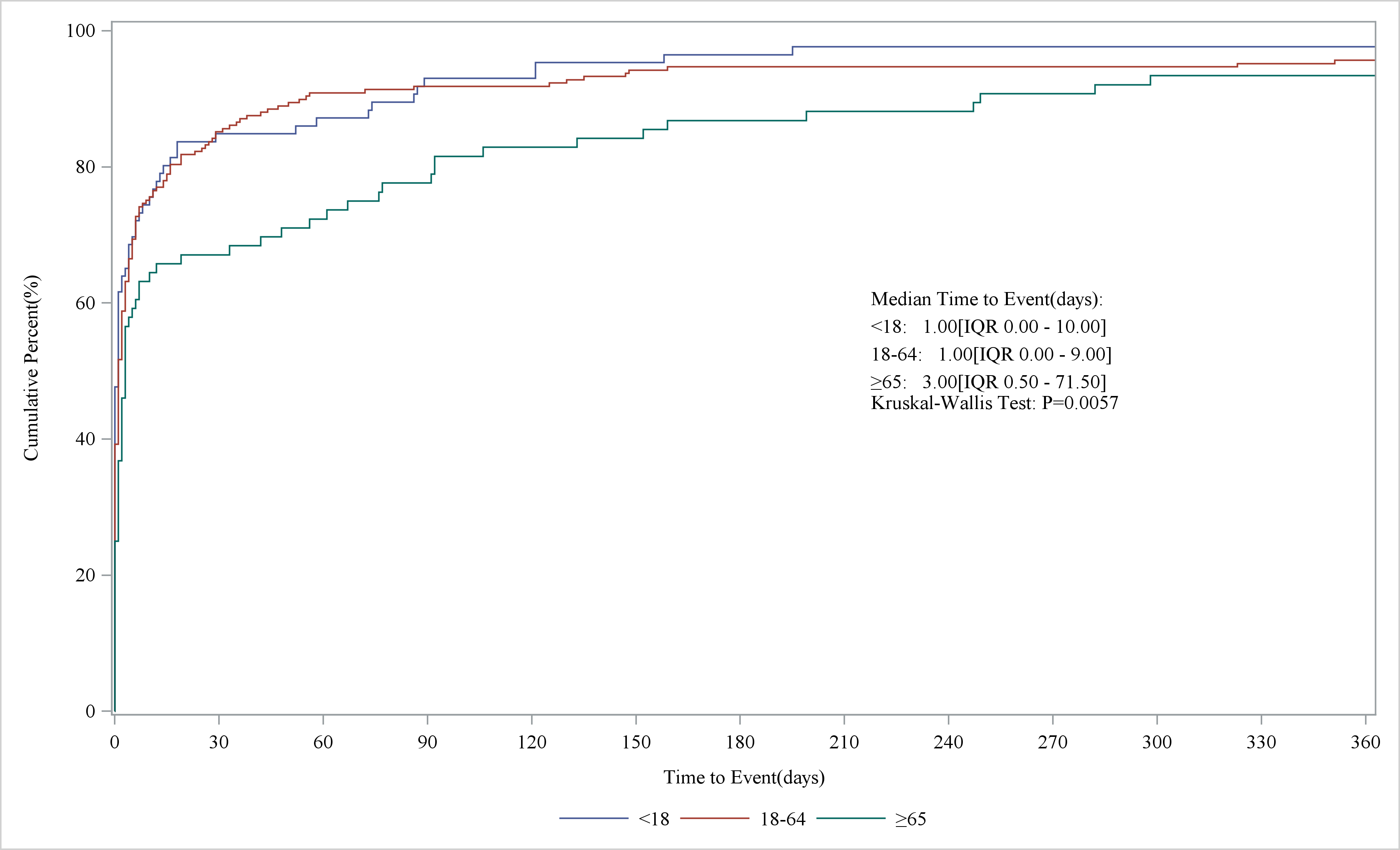


**Figure S5.** Time to onset of neuropsychiatric ADEs for cetirizine and levocetirizine in FAERS. (A) Time to onset for cetirizine, stratified by sex. (B) Time to onset for levocetirizine, stratified by sex. (C) Time to onset for cetirizine, stratified by age group. (D) Time to onset for levocetirizine, stratified by age group. (F: Female, M: Male, pediatric: ≤18 years, adult: 19–64 years, elderly: ≥65 years).

**References:**

[1] Kumar A. The Newly Available FAERS Public Dashboard: Implications for Health Care Professionals. Hosp Pharm 2019;54(2):75-7.<http://doi.org/10.1177/0018578718795271>.

[2] Agency EM. EurdraVigilance System Overview 2024 [2025-11-14]. Available from: <https://www.ema.europa.eu/en/human-regulatory-overview/research-development/pharmacovigilance-research-development/eudravigilance/eudravigilance-system-overview>.

[3] Agency EM. Guideline on good pharmacovigilance practices (GVP) — Module VI – Collection, management and submission of reports of suspected adverse reactions to medicinal products (Rev 2) 2017 [2025-11-14]. Available from: <https://www.ema.europa.eu/en/documents/regulatory-procedural-guideline/guideline-good-pharmacovigilance-practices-gvp-module-vi-collection-management-and-submission-reports-suspected-adverse-reactions-medicinal-products-rev-2_en.pdf>.

**Appendix 1. READUS-PV Checklist**

**READUS-PV Checklist for reporting disproportionality reporting rate investigations (main manuscript).**

| **Section and topic** | **Item #** | **Checklist item** | **Location where item is reported** |
| --- | --- | --- | --- |
| **Title** |  |  |  |
|  | *1a* | *If disproportionality analyses are a prominent component of the published study, the study should be identified as a “disproportionality analysis”. The type of data and name of the database(s) should be specified.* | *P. 1* |
|  | *1b* | *Report the name of adverse event(s) and/or drug(s) under study, when applicable.* | *P. 1* |
| **Introduction** |  |  |  |
| Background | *2a* | *Describe the drug(s) and its utilization, the nature of the adverse event(s) under study and its frequency, and the existing knowledge on the drug-event combination.* | *Background Para. 1* |
|  | *2b* | *Specify the rationale for performing the analysis, e.g., as part of routine pharmacovigilance, to investigate an overall safety profile, or to assess a pre-specified hypothesis.* | *Background Para. 1-2* |
|  | *2c* | *Explain why ICSR databases and disproportionality analysis are suitable to fill the knowledge gap.* | *Background Para. 3* |
| Objectives | *3* | *State specific objectives, identifying the adverse event(s), the drug(s), and the reference group, including any pre-specified hypothesis, if applicable.* | *Background Para. 4* |
| **Methods** |  |  |  |
| Study design | *4a* | *Identify the study (i.e., “disproportionality analysis”) and the type of data used (e.g., “individual case safety reports”).* | *Methods Para. 1* |
|  | *4b* | *Provide an outline of the entire study design, including primary and sensitivity analyses performed, and other designs such as case-by-case analysis or literature review.* | *Figure 1* |
| Data description, access, and pre-processing | *5a* | *Specify the name of the database(s), the database(s) custodian, and the coverage. Specify the type/number of drugs included within the database and the thesaurus, taxonomies, or ontologies used for coding drugs and events.* | *Methods Para. 1-4 +Figure 1* |
|  | *5b* | *Specify the extraction dates and describe and justify all choices used for data pre-processing, including any data transformation or exclusion, if appropriate.* | *Methods Para. 1-4 +supplementary P. 1* |
| Variables definition | *6a* | *Describe the study population, including any restriction.* | *Figure 1* |
|  | *6b* | *Describe the nature and the meaning of key variables assessed in the work.* | *Methods Para. 4* |
|  | *6c* | *Specify and justify any grouping of drugs or events. For drugs, specify and justify whether active ingredients/trade names/salts were considered and/or the selected role.* | *Methods Para. 3, 7* |
|  | *6d* | *Describe any additional data source used, the type of data, and how they interact with ICSRs.* | *NA* |
| Statistical methods | *7a* | *Present any descriptive analysis performed, specifying variables investigated, statistical tests, and significance thresholds.* | *NA* |
|  | *7b* | *Describe the measure(s) selected for the disproportionality analysis including any threshold used to identify signals of disproportionate reporting. Explain the reason for this choice if applicable.* | *Methods Para. 5 + Supplementary table 1* |
|  | *7c* | *Clearly describe any sensitivity analysis and any tool to control confounding, including any restriction, subgroup, stratification, adjustment, or interaction.* | *Sections 2.3 and 2.4* |
|  | *7d* | *Specify the variables and methods used for the case-by-case analysis, including any algorithm or criteria used to assess causality, if performed.* | *NA* |
|  | *7e* | *Specify any statistical methods used for other data sources.* | *NA* |
| **Results** |  |  |  |
| Participants | *8a* | *Specify the number of individual case safety reports included at each stage, including reasons for exclusion.* | *Figure 1* |
|  | *8b* | *Provide key demographic and clinical characteristics of cases, if possible comparing cases with any appropriate reference group.* | *Section 3.1* |
| Disproportionality analysis | *9* | *Present all results including confidence intervals. Present also results of sensitivity analyses, if performed.* | *Results Section 3.2-3.5* |
| Case-by-case analysis | *10* | *Present the case-by-case analysis of key variables. Present the causality assessment, if applicable.* | *NA* |
| **Discussion** |  |  |  |
| Key results | *11* | *Discuss key results with reference to study objectives and contextualize them within the current literature and other consulted sources. Clearly discriminate between expected reactions and emerging safety signals.* | *Discussion Para. 1-10* |
| External validity | *12a* | *Discuss the external validity of the results to the general population.* | *Discussion Para. 1-10* |
|  | *12b* | *Discuss the potential relevance of results in clinical practice* | *Discussion Para. 1-10* |
|  | *12c* | *Propose further study designs if applicable* | *NA* |
| Limitations | *13* | *Present general limitations, making clear that disproportionality analysis alone cannot prove causation or measure incidence, and specific limitations, including confounding and reporting bias and efforts to mitigate them.* | *Section Limitations* |
| **Declarations** |  |  |  |
|  | *14a* | *Provide the source of funding/sponsorship and the role of the funders/sponsors for the present study and for any original study on which the present article is based.* | *Section Funding* |
|  | *14b* | *Clearly identify potential commercial and intellectual conflicts of interest (e.g., link to any drug/event investigated, whether financial, legal action, or software used).* | *NA* |
|  | *14c* | *Declare any institutional approval needed or granted in the investigation.* | *NA* |
|  | *14d* | *Include a statement on data availability, code availability (including the version of the statistical software used), and protocol registration.* | *Section Availability of data and materials* |

**The READUS-PV checklist for abstracts**

| **Section and topic** | **Item #** | **Checklist item** | **Location where item is reported** |
| --- | --- | --- | --- |
| Background | *1a* | *State the aim/rationale for performing the study.* | *Para. 1* |
|  | *1b* | *Specify the adverse event(s) and/or the drug(s) under study, when applicable.* | *Para. 1* |
|  | *1c* | *Specify the specific population or setting, when applicable.* | *Para. 1* |
| Methods | *2a* | *Identify the study as a “disproportionality analysis” and specify the type of data used.* | *Para. 2* |
|  | *2b* | *Specify the name of the database(s) used and the type of access.* | *Para. 2* |
|  | *2c* | *Specify the timeframe and geographical region, when applicable.* | *Para. 2* |
|  | *2d* | *Specify the disproportionality measure(s) used and their statistical significance threshold(s).* | *Para. 2* |
|  | *2e* | *Specify if a case-by-case analysis is performed.* | *NA* |
| Results | *3* | *Report main findings including their precision (e.g., 95% confidence intervals), together with a short summary of the case-by- case analysis.* | *Para. 3* |
| Conclusion | *4a* | *Clearly report key conclusions.* | *Para. 4* |
|  | *4b* | *Acknowledge that the disproportionality analysis is a hypothesis generating or refinement approach.* | *NA* |
|  | *4c* | *State the implications and clinical relevance of the findings.* | *Para. 4* |

**Appendix 2: code**

**FAERS data deduplication code**

data DEMO;

set alldata.DEMO;

fda_dtn=input(fda_dt,??yymmdd10.);

run;

proc sort data=demo out= demo sortseq=linguistic(numeric_collation=on);by caseid fda_dtn primaryid ;quit;

data demo;

set demo;

by caseid fda_dtn primaryid;

if last.caseid then output;

drop fda_dtn;

run;

proc sort data=Demo(keep= primaryid caseid GetDataYear GetDataQT) out=temp1 dupout=a1_3 nodupkey;

by primaryid GetDataYear GetDataQT;

run;

data raw.primaryid;

set temp1;

by primaryid GetDataYear GetDataQT;

if last.primaryid then output;

run;

proc sql UNDO_POLICY=NONE;

create table raw.Delprimaryid as

select distinct *

from raw.primaryid where caseid in (select VAR1 from alldata._delete)

;

quit;

proc sql UNDO_POLICY=NONE;

create table raw.primaryid as

select distinct *

from raw.primaryid where caseid not in (select VAR1 from alldata._delete)

;

quit;

proc sql UNDO_POLICY=NONE;

create table raw.primaryid as

select distinct *

from raw.primaryid where strip(primaryid)||"-"||strip(put(GetDataYear,z2.))||"-"||strip(put(GetDataQT,z2.)) in (select strip(primaryid)||"-"||strip(put(GetDataYear,z2.))||"-"||strip(put(GetDataQT,z2.)) from alldata.Reac_code) ;

quit;

%macro selectdata1(outds=,fromds=);

proc sql UNDO_POLICY=NONE;

create table &outds. as

select *

from &fromds. where strip(primaryid)||"-"||strip(put(GetDataYear,z2.))||"-"||strip(put(GetDataQT,z2.)) in (select strip(primaryid)||"-"||strip(put(GetDataYear,z2.))||"-"||strip(put(GetDataQT,z2.)) from raw.primaryid) ;

quit;

%mend;

%selectdata1(outds=raw.DRUG,fromds=alldata.DRUG);

%selectdata1(outds=raw.Reac_code,fromds=alldata.Reac_code);

%selectdata1(outds=raw.DEMO,fromds=alldata.DEMO);

%selectdata1(outds=raw.RPSR,fromds=alldata.RPSR);

%selectdata1(outds=raw.THER,fromds=alldata.THER);

%selectdata1(outds=raw.OUTC,fromds=alldata.OUTC);

%selectdata1(outds=raw.INDI,fromds=alldata.INDI);

%selectdata1(outds=raw.Indi_code,fromds=alldata.Indi_code);

**Disproportionality analysis code**

data want;

set have;

/*************************

ROR

***************************/

ROR=(a*d)/(b*c);

RORL=exp(log(ROR)-1.96*sqrt(1/a+1/b+1/c+1/d));

RORU=exp(log(ROR)+1.96*sqrt(1/a+1/b+1/c+1/d));

if a>=3 and RORL>1 then RORYN="Y";

else RORYN="N";

label ROR="ROR" RORL='95%CI Lower' RORU=95%CI Upper' RORYN="ROR（Y，N）";

/*************************

PRR

***************************/

CHIQ2=(((a*d-c*b)**2)*(a+b+c+d))/((a+b)*(c+d)*(a+c)*(b+d));

CHIQ1=(((abs(a*d-c*b)-(n/2))**2)*N)/((a+b)*(c+d)*(a+c)*(b+d));

if a>=3 and CHIQ2>=4 and PRR>=2 then MHRAYN="Y";

else MHRAYN="N";

label CHIQ2="Chi-Square" PRR="PRR（Y，N）";

drop CHIQ1;

/*************************

BCPNN

***************************/

alpha1=1;

bate1=1;

alpha=2;

bate=2;

R11=1;

Cx=a+b;

Cy=a+c;

Cxy=a;

IC =log2((a*(a+b+c+d))/((a+b)*(a+c)));

r=R11*(((N+alpha)*(N+bate))/((Cx+alpha1)*(Cy+bate1)) );

EIC=log2(( (Cxy+R11)*(N+alpha)*(N+bate) )/((N+R)*(Cx+alpha1)*(Cy+bate1)));

VIC_Part1= (N-Cxy+r-r11)/((Cxy+R11)*(1+N+r));

VIC_Part2= (N-Cx+alpha -alpha1)/((Cx+alpha1)*(1+N+alpha));

VIC_Part3= (N-Cy+bate-bate1)/((Cy+bate1)*(1+N+bate));

VIC_Part4= (log(2))**2;

VIC=(VIC_Part1+VIC_Part2+VIC_Part3)/VIC_Part4;

EIC_L=EIC-(2*sqrt(VIC));

EIC_U=EIC+(2*sqrt(VIC));

if EIC_L>0 then ICYN="Y";

else ICYN="N";

if EIC_L<=0 then ICYN2=cat("(-)");

else if 0<EIC_L<=1.5 then ICYN2=cat("(+)");

else if 1.5<EIC_L<=3 then ICYN2=cat("(++)");

else if 3<EIC_L then ICYN2=cat("(+++)");

label IC="IC" EIC="EIC" EIC_L='IC（95%CI Lower/IC025）'

EIC_U='IC（95%CI Upper）'

ICYN="IC（Y，N）"

ICYN2="IC"

;

drop VIC_Part1 VIC_Part2 VIC_Part3 VIC_Part4 alpha1 bate1 alpha bate R11 Cx Cy Cxy R VIC ;

/*************************

MGPS

***************************/

EBGM=(a*(a+b+c+d))/((a+c)*(a+b));

EBGML=exp(log(EBGM)-1.96*sqrt(1/a+1/b+1/c+1/d));

EBGMU=exp(log(EBGM)+1.96*sqrt(1/a+1/b+1/c+1/d));

if EBGML>2 then EBGMYN='Y';

else EBGMYN='N';

label EBGML ='EBGM（95%CI Lower）' EBGMU='EBGM（95%CI Upper）' EBGM="EBGM" EBGMYN="EBGM（Y，N）" ;

label a="a"

b="b"

c="c"

d="d"

N="N" ;

format ROR RORL RORU PRR PRRL PRRU CHIQ2 VIC EIC IC EIC_L EIC_U EBGM EBGML EBGMU 7.2;

run;
